# Discovery of an autophagy inducer J3 to lower mutant huntingtin and alleviate Huntington’s disease-related phenotype

**DOI:** 10.1186/s13578-022-00906-3

**Published:** 2022-10-08

**Authors:** Jiahui Long, Xia Luo, Dongmei Fang, Haikun Song, Weibin Fang, Hao Shan, Peiqing Liu, Boxun Lu, Xiao-Ming Yin, Liang Hong, Min Li

**Affiliations:** 1 School of Pharmaceutical Sciences, Guangdong Provincial Key Laboratory of Chiral Molecule and Drug Discovery, National and Local Joint Engineering Laboratory of Druggability and New Drugs Evaluation, Guangdong Engineering Laboratory of Druggability and New Drugs Evaluation, Sun Yat-Sen University, Guangzhou, 510006 Guangdong China; 2 Greater Bay Area Institute of Precision Medicine (Guangzhou), Fudan University, Guangzhou, 511458 Guangdong China; 3 School of Life Sciences, Fudan University, Shanghai, 200438 China; 4 Department of Pathology and Laboratory Medicine, School of Medicine, Tulane University, New Orleans, LA 70112 USA

**Keywords:** Autophagy, HdhQ140, Huntington’s disease, J3, mHTT

## Abstract

Huntington’s disease (HD) is a neurodegenerative disorder caused by aggregation of the mutant huntingtin (mHTT) protein encoded from extra tracts of CAG repeats in exon 1 of the HTT gene. mHTT proteins are neurotoxic to render the death of neurons and a series of disease-associated phenotypes. The mHTT is degraded through autophagy pathway and ubiquitin–proteasome system (UPS). This study identified a small molecule, J3, as an autophagy inducer by high-content screening. The results revealed that J3 could inhibit mTOR, thus promoting autophagic flux and long-lived protein degradation. Further, J3 selectively lowered the soluble and insoluble mHTT but not wild type HTT levels in cell models. The HdhQ140 mice showed reduced HD-associated activity and loss of motor functions. However, administration of J3 showed increased activity and a slight improvement in the motor function in the open-field test, balance beam test, and rotarod tests. Furthermore, in vivo studies revealed that J3 decreased T-HTT and misfolded protein levels in the striatum and increased the levels of the medium spiny neuron marker DARPP-32. In addition, J3 showed good permeability across the brain-blood barrier efficiently, suggesting that J3 was a promising candidate for the treatment of HD.

**Supplementary Information:**

The online version contains supplementary material available at 10.1186/s13578-022-00906-3.

## Introduction

Neurodegenerative disorders, such as Alzheimer’s disease (AD), Parkinson’s disease (PD), and polyglutamine (polyQ) expansion diseases, occurs due to dysfunction of protein homeostasis characterized by aggregation of misfolded proteins, such as β-amyloid, α-synuclein, and mutant huntingtin (mHTT) [1, 2]. Huntington’s disease (HD) is one of the nine polyQ diseases. It is characterized by motor dysfunctions, memory impairment and disordered mental status [3, 4]. Huntington’s disease (HD) is an autosomal dominant neurodegenerative disorder that occurs due to abnormal expansion of the CAG repeat sequence in the exon 1 of the HTT gene. When CAG repeats exceed 35, the gene encodes aggregation-prone mHTT, which has an extra extended N-terminal polyQ tract and is highly prone to aggregation [2–4]. mHTT is toxic to neurons and impairs neuronal transmission as it induces endoplasmic reticulum stress, mitochondrial stress and oxidative stress [5]. Studies have shown that neuronal toxicity occurs due to the soluble mHTT [6, 7]. Further, toxic mHTT is associated with a HD-associated phenotypes. Protein aggregates (inclusion bodies) of mHTT cause deterioration, atrophy, and death of the striatal neurons [4].

Enhanced autophagy and the ubiquitin proteasome system (UPS) play important roles in the clearance of mHTT [8–10]. However, in HD, both UPS and autophagy pathways are inhibited, leading to decreased clearance and accumulation of mHTT. Recently, proteolysis-targeting chimeras (PROTACs) have been designed to degrade the target proteins via the UPS. This process is started by linking the target protein to an E3 ligase [11]. In neurodegenerative diseases, the designed PROTAC molecules showed limited effectiveness in clearing mHTT, as misfolded proteins tend to be degraded by autophagy. Autophagy clears macromolecules, including cell organelles and other large protein aggregates. The autophagy receptor CCT2 associates with aggregation-prone proteins independent of cargo ubiquitination and interacts with the protein microtubule-associated protein 1A/1B light chain 3 (LC3) to mediate the clearance of solid protein aggregates, including mHTT. Therefore, targeting autophagy in HD offers a more promising therapeutic strategy [12]. Compounds that interact with LC3 and the disease-causing protein may target the latter for autophagic clearance [13, 14]. Furthermore, in vitro and in vivo studies have shown that autophagy activators, including rapamycin, lithium chloride, and trehalose, could be used to treat HD as they lower mHTT levels [13, 15–18]. However, the use of these molecules is limited by low druggability. Therefore, there is a need to develop novel agents with high druggability for treating HD.

A previous study established a high content screening (HCS) assay based on GFP-LC3 puncta formation in mouse embryonic fibroblasts (MEF) cells, which screened out 50 autophagy modulators from a chemical library with about 20,000 compounds [19]. The study revealed that J3 could markedly induce GFP-LC3 puncta. In this study, we further identified that J3 could induce autophagy through mTOR inhibition. Besides, J3 could also selectively clear mHTT via both UPS and autophagy pathway in vitro. We further demonstrated that J3, with high permeability to the brain-blood barrier, could significantly alleviate HD-associated phenotypes and biomarkers, such as total HTT (T-HTT) and DARPP-32 in the mouse model of HD. Thus we discovered a novel small molecule J3 which could be a potent chemical for future HD treatment.

## Results

### J3 is a novel autophagy inducer

**Fig. 1:**
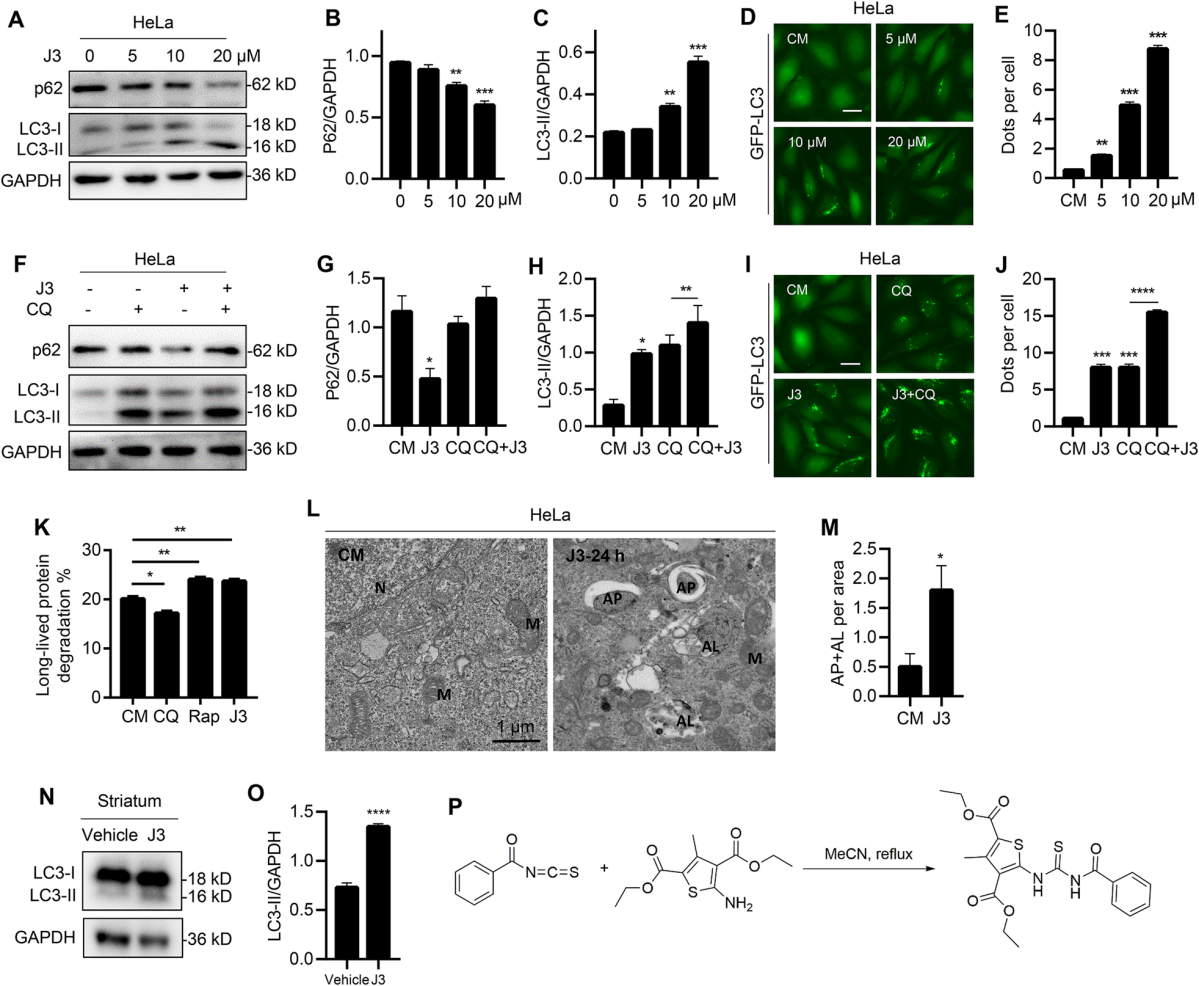
Identification of a novel autophagy inducer J3. **A** WT-HeLa cells were treated with 5, 10, 20 μM of J3 for 6 h, then p62 and LC3 were measured by immunoblotting. **B**, **C** The quantification of P62 (**B**) and LC3 (**C**) levels at different doses were analyzed. **D** HeLa cells stably expressing GFP-LC3 were incubated with 5, 10, 20 μM of J3 in complete medium (CM) for 6 h, then GFP-LC3 dots were analyzed. Scale bar = 20 μm. **E** GFP-LC3 dots from at least 50 cells were counted for quantification of different groups. **F** WT-HeLa cells were treated with 20 μM of J3 with or without 40 μM of chloroquine (CQ) for 6 h, then p62 and LC3 were analyzed by immunoblotting. **G**–**H** Quantification of P62 (**G**) and LC3 (**H**) levels of each group were analyzed. **I** GFP-LC3-HeLa were cultured and treated as **F**. **J** Quantification of GFP-LC3 dots was performed. **K** Hela cells were treated with 1 μM of rapamycin (Rap), 20 μM of CQ, and 10 μM of J3 for 16 h. Long-lived protein degradation assay was performed. **L** CFP-103Q-HeLa was treated with 20 μM of J3 for 24 h, the autophagosomes and autolysosomes were observed by transmission electron microscopy. *N* nucleus, *M* mitochondria, *AP* autophagosome, *AL* autolysosome. **M** Quantification of total AP plus AL per area was performed. Ten areas were analyzed. **N** Three 10-week-old C57BL/6 mouse were intraperitoneal injected with 30 mg/Kg of J3 daily, after 3 days, mice were sacrificed and the striatum were isolated for western blot analysis. **O** Quantification of LC3-II protein levels in **N** were analyzed. **P** The scheme of the one-step synthesis of J3. Data are presented as mean ± sem from three individual experiments or three different fields. *p < 0.05, **p < 0.01, ***p < 0.001

A previously conducted HCS revealed that J3 could induce autophagy [19]. The present study revealed that J3 induced LC3-II and decreased the expression of p62 in a dose-dependent and time-dependent manner (Fig. 1A–C and Additional file 1: S1A–C). In addition, J3 induced GFP-LC3 dots accumulation in HeLa, MEF, and A549 cells (Fig. 1D–E and Additional file 1: S1D–G). Different cells were treated with J3 with or without the lysosomal inhibitor, chloroquine (CQ), to confirm whether J3 was an autophagy inducer. HeLa and glioblastoma (U251) cell lines treated with both J3 and CQ showed increased accumulation of LC3-II or GFP-LC3 compared to those treated with CQ only, indicating that J3 was an autophagic inducer (Fig. 1F–J and Additional file 1: S1H–J). Further, we conducted the long-lived protein degradation assay. As shown in Fig. 1K and Additional file 1: Figure S1K, J3 enhanced the long-lived protein degradation similar to other well-known autophagy inducers, such as EBSS and Rap. The electron microscopy revealed that J3-treated CFP-103Q-HeLa cells had increased numbers of the autophagosomes and autolysosomes (Fig. 1L–M). The western blot analysis showed increased expression of LC3-II in the striatum, consistent with the in vitro study (Fig. 1N, O), indicating that J3 could activate autophagy in vivo. Taken together, this study revealed that J3 was an autophagy inducer. Further, high-purity synthesis of J3 was carried out using a one-step method (Fig. 1P) for further pharmacological studies.

### J3 inhibits the mTOR pathway

**Fig. 2:**
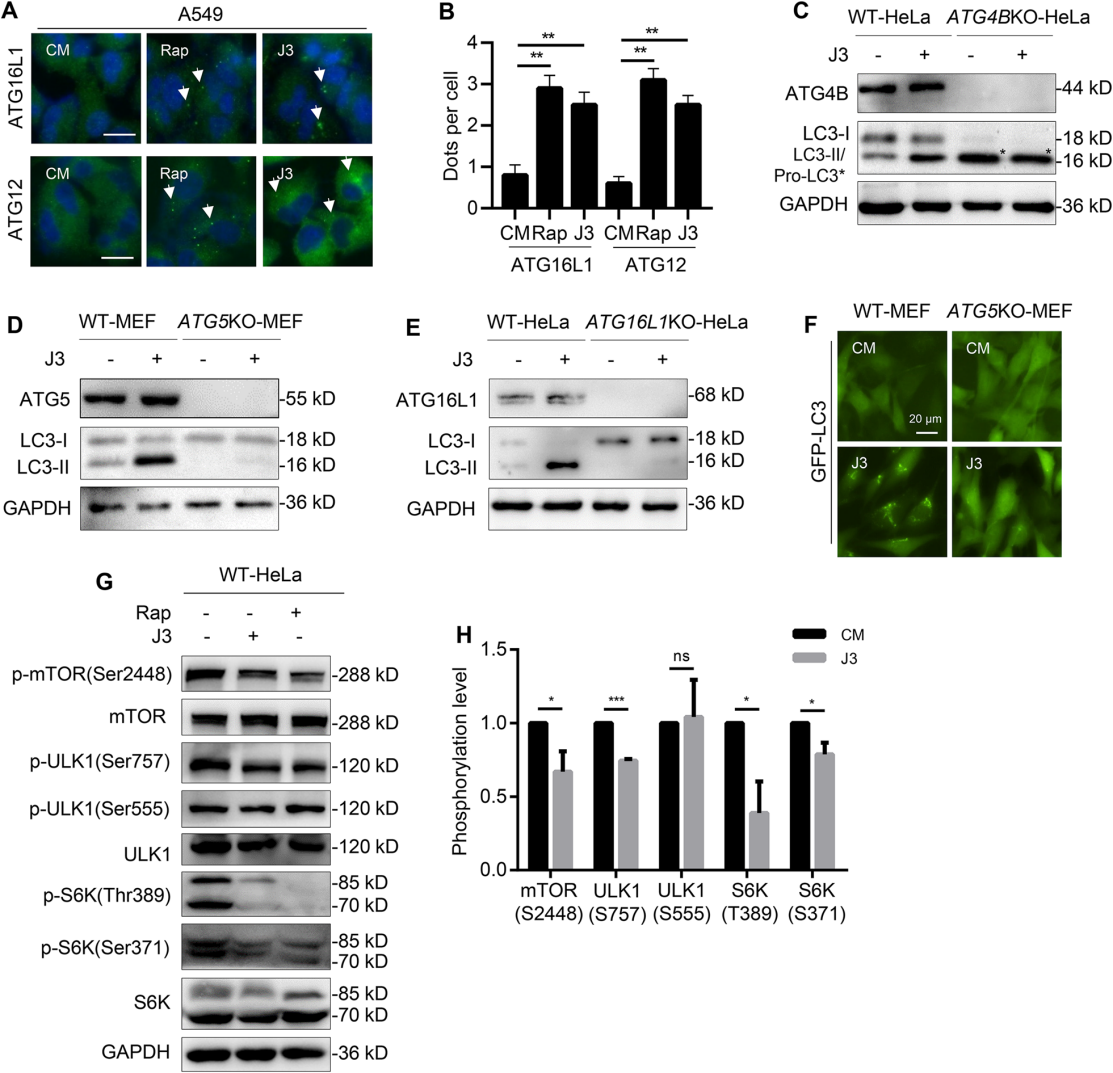
J3 inhibits mTOR pathway. **A** WT-A549 were treated with 20 μM of J3 or 1 μM of Rap for 6 h followed by immunostaining of ATG16L1 and ATG12. ATG16L1 and ATG12 positive structures were indicated by arrowhead. Scale bar = 20 μm. **B** ATG16L1 or ATG12 dots from at least 50 cells were counted for quantification of different groups. **C** WT and *ATG4B-*deficient HeLa cells were incubated with 20 μM of J3 for 6 h, then ATG4B, LC3-I, LC3-II (in WT-HeLa), and pro-LC3 (in *ATG4B-*deficient HeLa, with asterisk) were analyzed by immunoblotting. **D** WT and *ATG5-*deficient MEF cells were incubated with 20 μM of J3 for 6 h, then ATG5, LC3-I, and LC3-II were analyzed by immunoblotting. **E** WT and *ATG16L1-*deficient HeLa cells were incubated with 20 μM of J3 for 6 h, then ATG16L1, LC3-I, and LC3-II were analyzed by immunoblotting. **F** WT- and *ATG5*KO-MEF cells stably expressing GFP-LC3 were treated by 20 μM of J3 for 6 h. The distribution of GFP-LC3 dots was analyzed. **G** WT-HeLa cells were treated with 20 μM of J3 or 1 μM of Rap for 6 h. The total protein level and the phosphorylation level of mTOR, ULK1, and S6K were analyzed by immunoblotting as indicated. **H** Cells were treated as **G**, then relative phosphorylation level of each protein was calculated. Data are presented as mean ± sem from three individual experiments. *p < 0.05, ***p < 0.001, ns means not significant

Phagophore or autophagosome formation involves several autophagy-associated proteins. The ATG5-ATG12/ATG16L1 complex acts as an E3 ligase of a ubiquitin-like conjugation system and is recruited to the phagophore membrane [20]. This study showed that ATG16L1 and ATG12 positive structures were present in A549 cells treated with J3 or rapamycin (Fig. 2A, B).The immunoblot analysis showed that J3 did not induce the formation of LC3-II in ATG5-deficient MEFs or ATG16L1-deficient HeLa cells (Fig. 2D, E). Furthermore, J3 could not induce the formation of LC3-II in ATG4B-deficient HeLa cells in which only unprimed LC3 (pro-LC3) was left (Fig. 2C). In addition, treatment with J3 led to an increased accumulation of GFP-LC3 dots in the wild-type MEFs but not the ATG5KO-MEFs (Fig. 2F). These findings revealed that J3-induced autophagy requires autophagy-associated proteins such as ATG4B, ATG5, and ATG16L1.

To explore how J3 could induce autophagy, we investigate the mammalian target of rapamycin (mTOR) pathway signaling. As shown in Fig. 2G, H, mTOR and its associated kinases can be dephosphorylated, activation downstream signaling pathways, such as unc-51-like kinase 1 (ULK1). In addition, the phosphorylation of ULK1 at Ser757 instead of Ser555 was inhibited, indicating that mTOR inhibition was involved in J3-induced autophagy. Further, we investigated whether J3 could induce autophagy through other pathways, such as the reactive oxygen species (ROS) [21], adenosine 5’-monophosphate-activated protein kinase (AMPK) pathway [22], and mitogen-activated protein kinase (MAPK) pathways [23]. As shown in Additional file 2: Figure S2, the ROS scavenger N-Acetyl-L-cysteine (NAC), AMPK inhibitor Compound C, MAPK inhibitor U0126, and calcium chelating agent BAPTA slightly altered LC3-II induced by J3. These results indicated that J3-induced autophagy was independent of the AMPK and MAPK pathways.

### J3 reduces mHTT levels in HD cell lines

**Fig. 3:**
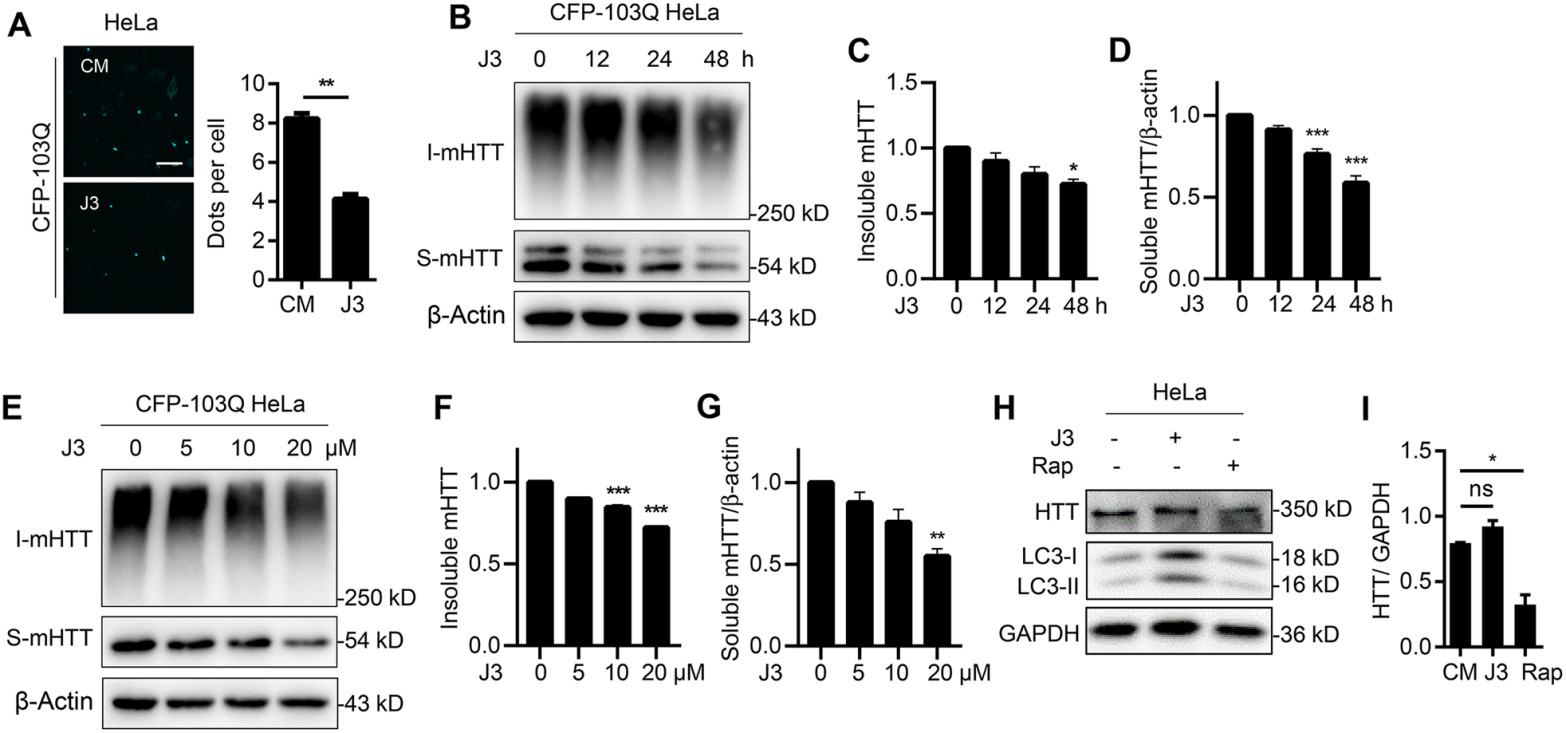
J3 reduces mHTT. **A** HeLa cells stably expressing CFP-103Q were treated with 20 μM of J3 for 48 h, the mHTT (disperse cyan fluorescence or aggregates) spots were counted from 100 cells, and quantified. Scale bar = 20 μm. **B** CFP-103Q-HeLa cells were treated with 20 μM of J3 for 12, 24, 48 h, respectively. The insoluble (I-mHTT) and soluble mHTT (S-mHTT) were detected by anti-GFP (sc-9996) for western blot. **C**–**D** The quantification of insoluble (**C**) and soluble mHTT (**D**) at different times was analyzed. **E** CFP-103Q-HeLa cells were treated with 5, 10, and 20 μM of J3 for 48 h, the protein level of mHTT (anti-GFP, sc-9996) were measured by western blot. **F**–**G** The quantification of insoluble (**F**) and soluble mHTT (**G**) treated by different doses of J3 was analyzed. **H** WT-HeLa cells were treated with 20 μM of J3 or 1 μM of Rap for 48 h, the endogenous HTT (anti-HTT, MAB2166) was measured by western blot. **I** The protein level of endogenous HTT of **H** was quantified. Data are presented as mean ± sem from three individual experiments or three different fields. *p < 0.05, **p < 0.01, ***p < 0.001, ns means not significant

To investigate whether J3 could decrease mHTT levels, we used CFP-103Q-HeLa cell lines that could stably express mHTT by replacing exon1 of HTT with exon1HTT carrying a polyQ expansion of 103 residues with the COOH terminal fused to monomeric enhanced CFP (mCFP) [24]. The results revealed that J3 significantly reduced dispersive cyan mHTT and mHTT aggregates-like mHTT significantly compared with complete medium group (Fig. 3A), suggesting that J3 could decrease mHTT. In order to precisely compare the protein level of insoluble fraction before and after J3 treatment, the insoluble fraction was quantified and separated by Tris–acetate polyacrylamide gels. We found J3 could reduce both soluble and insoluble mHTT in a dose- and time-dependent manner (Fig. 3B–G). Previous literature showed that the neurotoxicity of mHTT was mainly derived from the soluble one, which could render oxidative stress, mitochondria toxicity and cytotoxicity [6, 7]. In our study, the optimal dose and incubation time of J3 to decrease mHTT were primarily dependent on the degradation of both soluble and insoluble mHTT. So 20 μM and 48 h incubation of J3 were adopted in the following cellular experiments. Since electrophoresis using 3% Tris–acetate polyacrylamide gels is complicated to operate for protein separation, the total insoluble fractions collected as indicated without further quantification were directly loaded to 10% SDS-PAGE (Additional file 3: Fig. S3A–F). The simplified SDS-PAGE also revealed decreased levels of the soluble and insoluble mHTT following J3 treatment, suggesting both collection methods we used for insoluble fraction with or without further quantification are comparable for the measurement of mHTT. Moreover, we compared the effect of rapamycin and J3 on wild type HTT levels. As shown in Fig. 3H, I, treatment of WT-HeLa cells with J3 and rapamycin for 48 h revealed that rapamycin significantly reduced the HTT levels, while J3 did not alter HTT level (Fig. 3H, I). These results indicate that J3 decreases the soluble and insoluble fractions of mHTT in HD cell lines.

### J3 degrades mHTT through the UPS and autophagy pathways

**Fig. 4:**
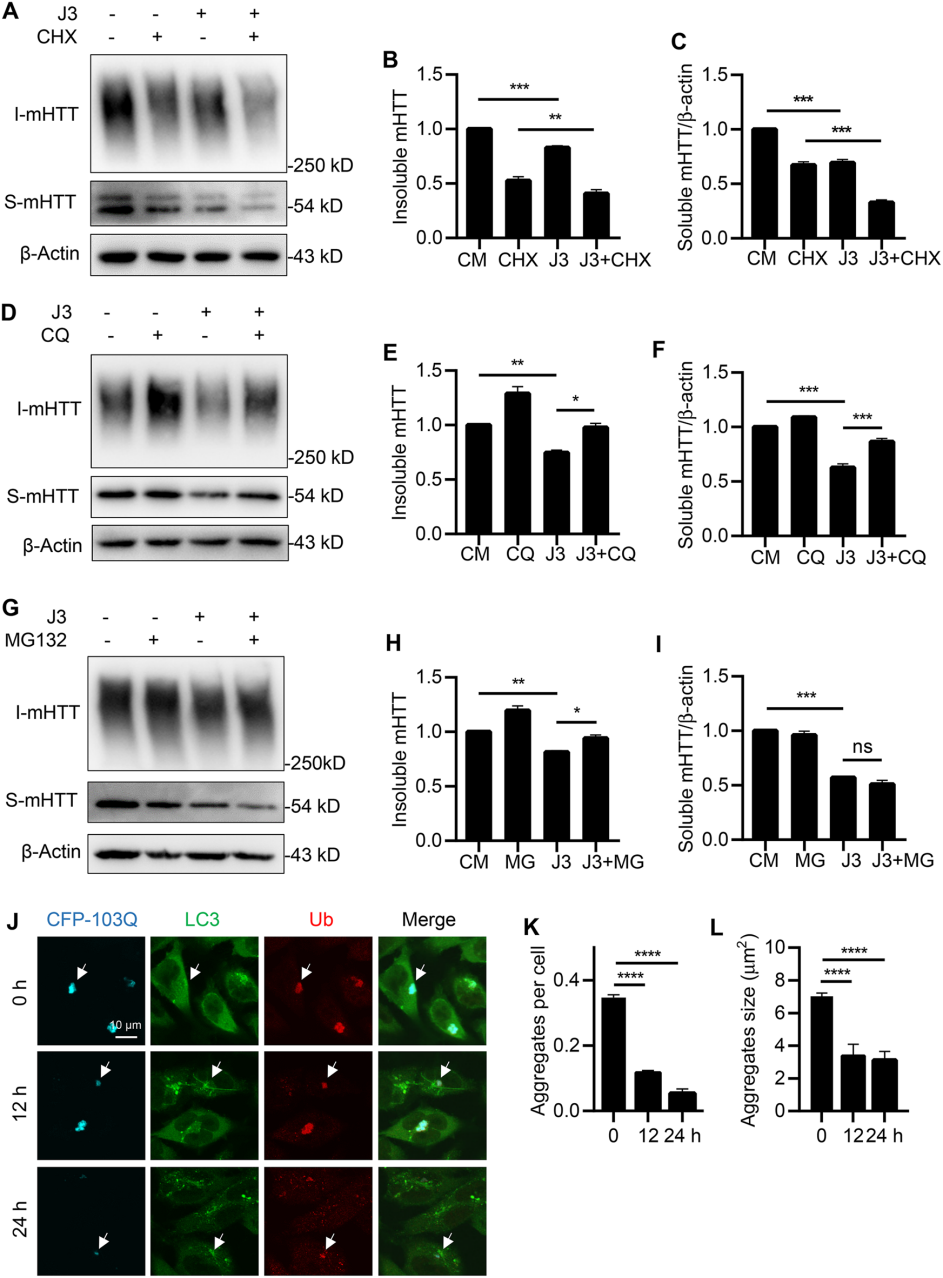
Both UPS and autophagy pathway were involved in mHTT clearance by J3. **A**–**C** CFP-103Q-HeLa cells were treated with 20 μM of J3 for 48 h. 10 μg/mL of CHX was added at the last 18 h. The protein expression of mHTT (anti-GFP, sc-9996) was detected by western blot (**A**), and the quantification of insoluble mHTT (**B**) and soluble mHTT (**C**) was analyzed. **D**–**F** CFP-103Q-HeLa cells were treated by 20 μM of J3 with or without 10 μM of CQ for 48 h. The protein expression of mHTT (anti-GFP, sc-9996) was detected by western blot (**D**), and the quantification of insoluble mHTT (**E**) and soluble mHTT (**F**) was analyzed. **G**–**I** CFP-103Q-HeLa cells were treated with 20 μM of J3 for 48 h. 5 μM of MG132 was added at the last 18 h. The protein expression of mHTT (anti-GFP, sc-9996) was detected by western blot (**G**), and the quantification of insoluble mHTT (**H**) and soluble mHTT (**I**) was analyzed. (**J**) HeLa cells stably expressing CFP-103Q (Cyan) were treated with J3 for 12 and 24 h, followed by immunostaining of endogenous Ub (Red) and LC3 (Green). Bar = 10 μm. **K**–**L** Quantification of aggregates number (**K**) and aggregates average size (**L**) was performed. Data are presented as mean ± sem from three individual experiments. *p < 0.05, **p < 0.01, ***p < 0.001, ns means not significant

The levels of proteins in cells could be reduced by decreasing protein synthesis or increasing protein degradation. This study showed that the protein synthesis inhibitor cycloheximide (CHX) inhibited the synthesis of soluble and insoluble fractions of mHTT. Further, treatment of cells with J3 and CHX resulted in a significant decrease in mHTT levels compared with treatment with CHX only (Fig. 4A–C). This finding suggested that J3-decreased mHTT levels mainly by increasing protein degradation. Moreover, treatment with J3 without CHX also reduced mHTT protein levels. Taken together, these findings indicate that J3 could promote the degradation of mHTT.

The autophagy-lysosome pathway and UPS are the two major protein degradation systems. Here, we used CQ, which can alkalize lysosomes to inhibit autophagy and MG132, which blocks the UPS to differentiate the degradation pathway. The results showed that CQ attenuated J3-induced degradation of the soluble and insoluble fractions of mHTT in CFP-103Q-HeLa (Fig. 4D–F). However, MG132 only attenuated J3-induced degradation of insoluble mHTT but exerted little effect on soluble mHTT (Fig. 4G–I). Degradation of mHTT proteins was also measured using simplified fractionation of mHTT without further quantification of the insoluble fraction (Additional file 4: Fig. S4A–I). Thus, these results suggested that J3 degrades mHTT via the autophagy-lysosome and UPS pathways.

In addition, the distribution of CFP-103Q, LC3, and Ub was analyzed by immunostaining. As shown in Fig. 4J–L, CFP-103Q aggregates were colocalized with LC3 and Ub before treatment with J3. However, upon treatment with J3, the aggregates were noted to be smaller. These results indicated that the autophagy and UPS pathways were crucial for mHTT clearance.

### The HD transgenic mouse shows moto dysfunction

**Fig. 5:**
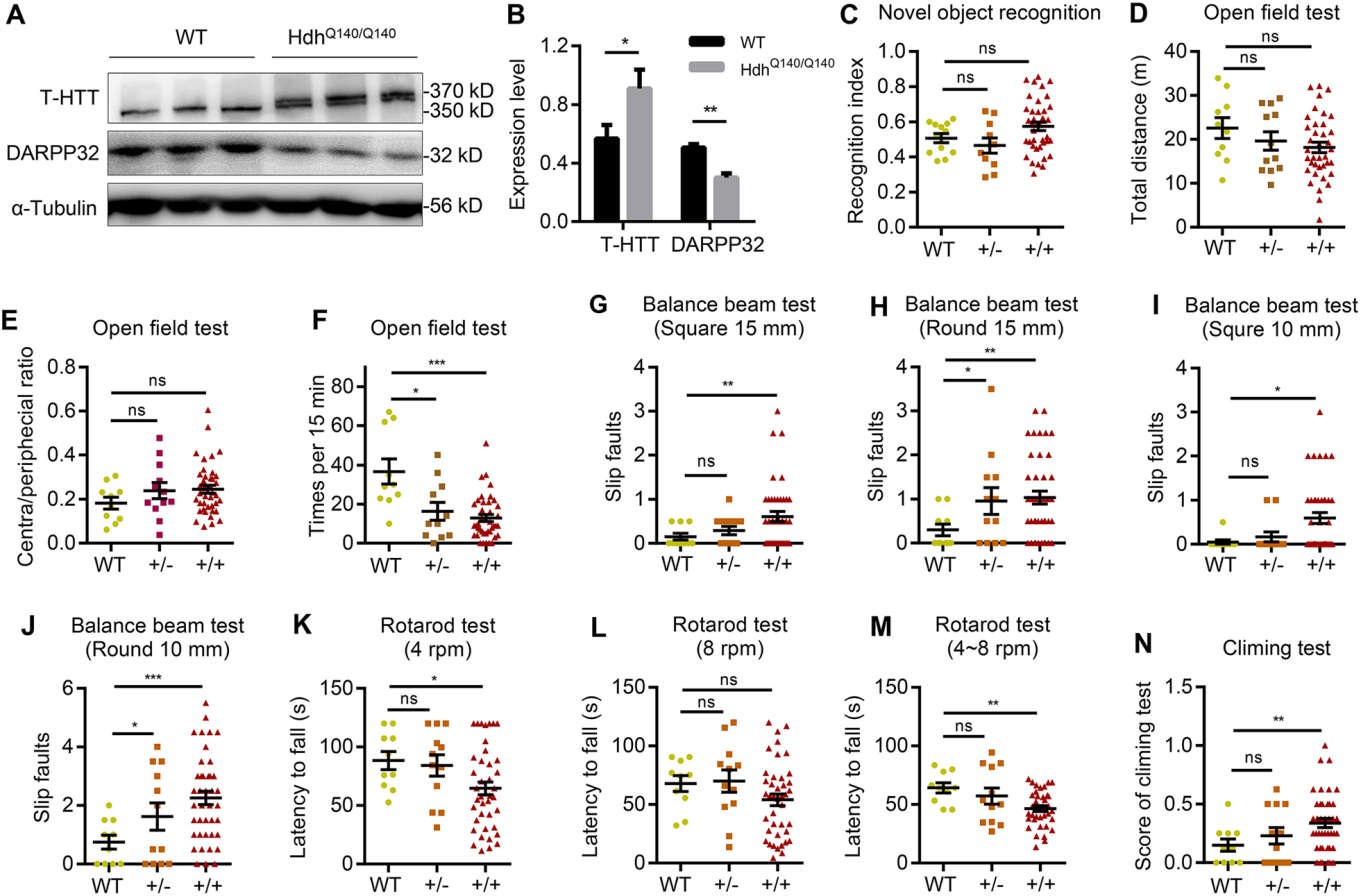
HD model Hdh^Q140^ transgenic mice appeared HD-associated biomarker and phenotypes. **A**–**B** The expression of T-HTT (anti-HTT, MAB2166) and DARPP-32 in the striatum of 12-month-old mice for homozygous Hdh^Q140/Q140^ (**A**), and the protein level were quantified (**B**). Data are presented as mean ± sem from three individual samples. **C** Recognition index of wild-type mice, heterozygote, and homozygous Hdh^Q140^ mice in novel object recognition test at 12-month-old. **D**–**F** Total distance (**D**), central /peripheral ratio (**E**), and times of lifting at least two limps in open-field test in 15 min (**F**) at 12-month-old. **G**–**J** Balance beam test of the slip faults in 15 mm square (**G**), 15 mm round (**H**), 10 mm square (**I**), and 10 mm round (**J**). **K**–**M** Rotarod assay of the latency time to fall at speed of 4 rpm (**K**), 8 rpm (**L**), 4 ~ 8 rpm (**M**) in 2 min. **N** The evaluation scores of mice in climbing test. Each dot represented an individual mouse, and bar represented mean ± SD. *p < 0.05, **p < 0.01, ***p < 0.001, ns means not significant

HD patients are characterized by motor impairment, cognitive disorders and mental disorders [25]. A knock-in mouse model was established by replacing exon1 with a mutant HTT exon1. This model (the HdhQ140 mouse model) is suitable for in vivo studies evaluating the related mechanisms of HD [26, 27]. First, we generated heterozygous and homozygous HdhQ140 mice as previously described (Additional file 5: Fig. S5A). The HdhQ140 mice revealed increased aggregation of mHTT in the striatal neurons, leading to the death of the medium spiny neurons and decreased striatal volume. The levels of dopamine and cAMP-regulated neuronal phosphoprotein 32 KDa (DARPP-32) were significantly reduced in the medium spiny neurons. The immunoblot analysis showed increased expression of T-HTT (anti-HTT, MAB2166) and decreased expression of DARPP-32 in the striatum of the heterozygous and homozygous HdhQ140 mice models (Fig. 5A, B, Additional file 5: Fig. S5D). Similarly, the immunostaining showed increased expression of T-HTT in the striatum of the 12-month-old homozygous HdhQ140 mice (Additional file 5: Fig. S5C).

Further, we conducted behavioral tests in 12-months-old-HdhQ140 mice to determine which gene type showed a more prominent HD-associated phenotype. Firstly, novel object recognition (NOR) was performed to analyze the cognitive functions of HdhQ140 mice [28]. As seen in Fig. 5C, there were insignificant differences in the recognition index between the WT mice and the heterozygotes or homozygous Hdh^Q140/Q140^ mice, indicating that the Hdh^Q140/−^ mice had little influence in cognitive functions. Furthermore, an open-field test conducted over 15 min showed slight differences in the total distance travelled and time spent in the inner and outer zones between the Hdh^Q140/−^, Hdh^Q140/Q140^ and WT mice (Fig. 5D, E). In the open-field test, mice standing on two limbs to explore was treated as an autonomous activity. The activity of mice was assessed by freely allowing the mice to explore for 15 min. As seen in Fig. 5F, the Hdh^Q140/−^ mice and the Hdh^Q140/Q140^ mice spent less time exploring compared with the WT mice, indicating that they were less active.

Furthermore, the balance beam, rotarod, and climbing tests were conducted to characterize mHTT-associated motor dysfunctions. The square balance beam tests revealed no significant differences in slip faults between the heterozygous Hdh^Q140/−^ mice and the WT mice. However, the slip faults in the Hdh^Q140/Q140^ mice were significantly increased (Fig. 5G). On the other hand, the round balance beam test showed increased slip faults in the Hdh^Q140/Q140^ and the Hdh^Q140/−^ groups (Fig. 5H). The 10 mm and 15 mm balance beam tests showed similar results (Fig. 5I, J). Similarly, the differences between the round and square balance beam tests for the Hdh^Q140/−^ mice and WT mice were not significant.

The mice were evaluated for balance and coordination in the rotarod test, which determined the latency time to fall off the rotarod. The Hdh^Q140/Q140^ mice showed a significant reduction of the latency time at 4 rpm and 4 ~ 8 rpm compared with Hdh^Q140/−^ mice (Fig. 5K–M). In the climbing test, the climbing performance of the mice was scored, with a higher score indicating poorer performance in motor ability. There were no significant differences in the climbing test between the WT and Hdh^Q140/140^ mice (Fig. 5N).

### J3 alleviates HD-associated phenotypes and biomarkers of Hdh^Q140/Q140^ mice

Acute toxicity testing was carried out to investigate the toxic effects of J3. A low and medium dose of 30 mg/kg and 60 mg/kg were given via intraperitoneal injection. All mice in the behavioral experiments were grouped by gene type, and mice showing abnormal behavior at 12 months were excluded. The behavioral tests were conducted as previously described. In brief, mice were allocated randomly into four groups (n = 10 mice in each group), including the vehicle group (WT), model group (Hdh^Q140/Q140^), low-dose group (Hdh^Q140/Q140^, J3 at 30 mg/kg), and medium-dose group (Hdh^Q140/Q140^, J3 at 60 mg/kg). Due to the similarity of behavior results between the low-dose and medium-dose groups, J3 at 30 mg/kg was used in subsequent experiments.

**Fig. 6:**
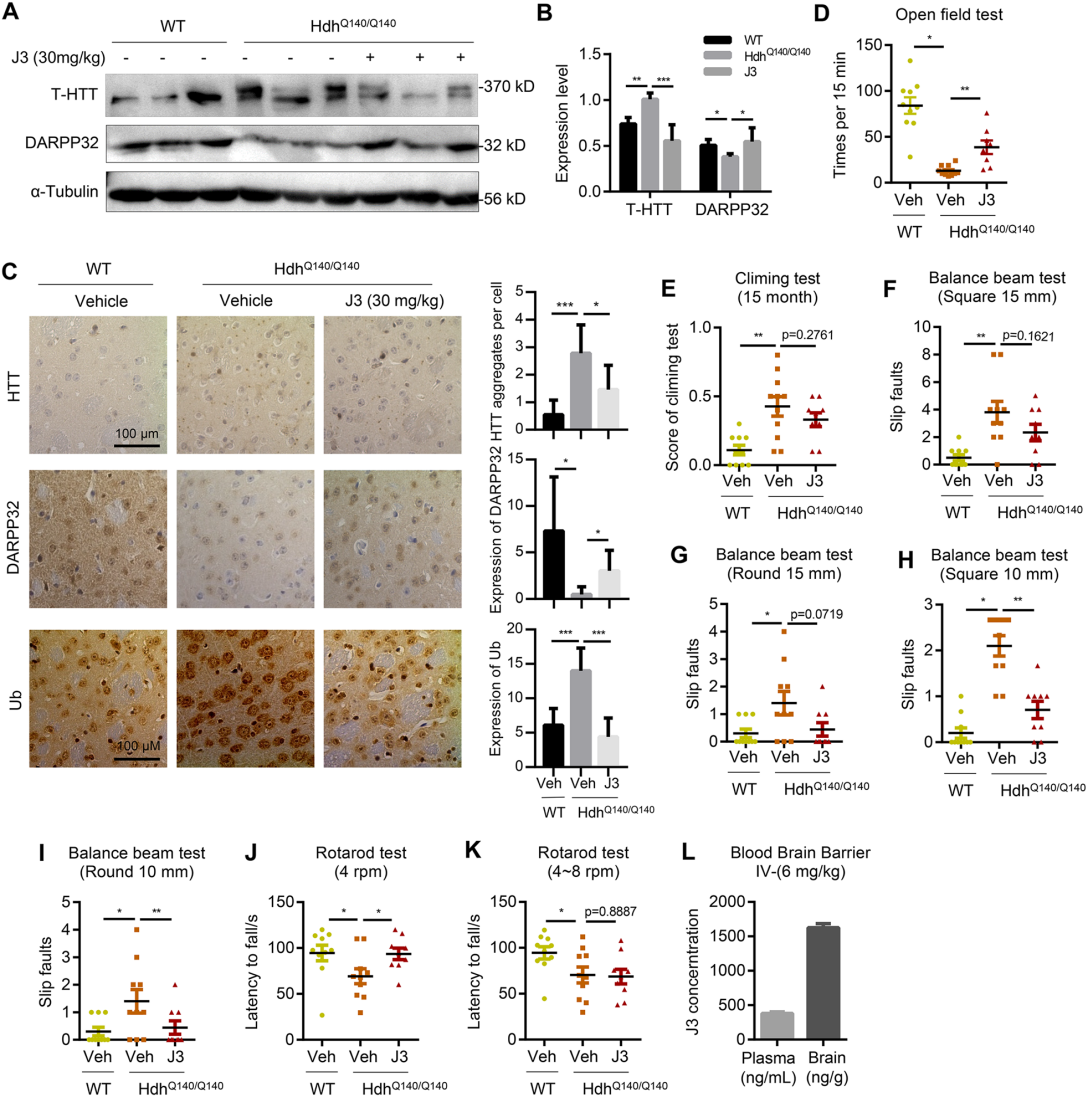
J3 alleviate HD-associated biomarker and phenotypes of the Hdh^Q140^ mouse model. **A**–**B** The expression of T-HTT (anti-HTT, MAB2166) and DARPP-32 in the striatum of 15-month-old mice for homozygous Hdh^Q140/Q140^ with or without 30 mg/kg of J3 administration for 3 months (**A**), and the protein level was quantified (**B**). Data are presented as mean ± sem from three individual samples. **C** The DAB staining of T-HTT (anti-HTT, MAB2166), DARPP-32, and Ub of striatum of indicating groups at 15-month-old. Protein expression level was quantified. Data are presented as mean ± sem from three individual samples. Scale bar = 100 μm. **D** Times of lifting at least two limps in open-field test for 15 min at 15-month-old for indicated groups. **E** The evaluation scores indicated phenotypes of the mouse in climbing test. **F**–**I** Balance beam test of the slip faults times of three types of mice in 15 mm square (**F**), 15 mm round (**G**), 10 mm square (H), and 10 mm round (**I**). (**J**–**K**) Rotarod test of the latency time of indicating groups at speed of 4 rpm (**J**), and 4 ~ 8 rpm (**K**) in 2 min. **L** 6 mg/kg of J3 was intravenous injected into 10-week-old mice. Plasma and brain concentrations of J3 were detected at 30 min. Each dot represented an individual mouse, and the bar represented mean ± SD. *p < 0.05, **p < 0.01, ***p < 0.001

HTT and DARPP-32 were expressed throughout the mouse brain. However, the main tissue marker changes of HD were seen in the striatum. Therefore, the striatum was isolated for the immunoassays (Additional file 5: Fig. S5B). The results revealed that administration of J3 at different doses for 12 weeks decreased the levels of T-HTT and DARPP-32 in Hdh^Q140/Q140^, indicating that HD changes could be ameliorated by J3 (Fig. 6A, B, Additional file 6: S6A-B). Further, the immunohistochemical staining results were consistent with the immunoblot analysis results (Fig. 6C). In neurodegenerative diseases, the degradation of misfolded proteins by the proteasome is inhibited, leading to the accumulation of ubiquitin. Therefore, we quantified the levels of ubiquitin to reflect levels of misfolded proteins. As shown in Fig. 6C, the Hdh^Q140/Q140^ mice showed increased accumulation of ubiquitin. However, treatment with J3 resulted in decreased accumulation of ubiquitin, suggesting that UPS pathway might be involved in the clearance of misfolded mHTT in vivo.

The behavioral tests conducted after administration of J3 (30 mg/kg) for 12 weeks showed decreased activity in the model group but increased activity in the vehicle group (Fig. 6D), demonstrating that J3 increased activity in the Hdh^Q140/Q140^ mice and improved the HD phenotypes. In the climbing test, the model group scored higher than the vehicle group. However, administration of J3 slightly decreased the score of the model group (Fig. 6E), indicating that J3 slightly improved motor function. All the balance beam tests showed increased slip faults in the model group suggesting a loss of motor function (Fig. 6F–I). The groups treated with J3 showed reduced slip faults in the 15 mm balance beam test. However, J3 did not reverse the loss of motor function (Fig. 6F, G). The 10 mm balance beam test showed a significant decrease in slip faults for the J3 treated groups (Fig. 6H, I). In the rotarod test, although administration with J3 did not fully reverse the loss of motor function at 4 ~ 8 rpm, there were slight improvements in motor functions at 4 rpm (Fig. 6J, K), demonstrating that J3 could ameliorate HD-associated motor impairment phenotypes.

### J3 has good blood–brain barrier (BBB) permeability

The physical and chemical properties of J3 should be considered when evaluating its druggability. The physicochemical parameters of J3 were determined using the ACD/Percepta software (ACD/Labs, Toronto, Canada). As shown in Additional file 6: Figure S6C, J3 is highly lipophilic and CNS-penetrant. The permeability of J3 across the BBB was determined using the LC–MS/MS method in ICR mice following a single intravenous injection. The results showed that the plasma concentration of J3 was 379.50 ng/mL while the concentration in the brain was 1626.02 ng/g at 0.5 h post-dose (Fig. 6L). These results suggested that J3 had a good permeability across the BBB and thus could be used in the treatment of neurodegenerative diseases.

## Discussion

Activation of autophagy results in decreased levels of toxic proteins, ameliorate signs of neurodegenerative disease and delays disease progression [29]. Some previous studies revealed that inducing autophagy with rapamycin or other small molecules could lower the cellular toxicity of mHTT [30–32]. Therefore, inducing autophagy prior to pathogenic insults offers a viable strategy for preventing disease onset.

In this study, a novel autophagy inducer J3 discovered by HCS was evaluated in HD. The results revealed that J3 could induce autophagy, stimulate LC3 lipidation, and promote the generation of autophagosomes. Further, the long-lived protein degradation assay revealed that J3 had a strong protein degradation ability. Moreover, J3 reduced the soluble and insoluble fractions of mHTT in CFP-103Q-Hela cells. Drug development for neurodegenerative diseases remains quite challenging. However, J3 showed good permeability across the BBB. In addition, the acute toxicity tests revealed that J3 had a low toxicity profile. Therefore, J3 is a promising drug for the treatment of neurodegenerative diseases.

Previous studies demonstrated that the UPS and autophagy pathways were ineffective in clearing mHTT, thus causing the accumulation of mHTT [33]. The present study revealed that J3 promoted the degradation of mHTT by inducing the UPS and autophagy pathways. Although mTOR inhibition is involved in autophagy initiation, the exact target of J3 in activating autophagy is still not clear. Furthermore, J3 increased the degradation of both the soluble and insoluble mHTT through autophagy. However, only the insoluble mHTT was degraded via the UPS pathway, suggesting that both pathways were involved in the clearance of mHTT. This finding could be explained by different possibilities (i) J3 could separately induce the autophagy and UPS pathways to increase the degradation of misfolded or aggregated mHTT. (ii) J3 could promote the ubiquitination of mHTT via different ubiquitin linkages, such as K48- or K63-linked ubiquitin chains [34]. The K48-mediated ubiquitination promotes mHTT degradation through proteasomes. On the other hand, mHTT aggregates could be cleared by K63-dependent autophagy mechanisms [35, 36]. mHTT is toxic to cells. However, wild-type HTT plays an important role in maintaining cellular homeostasis and embryonic development [37]. Therefore, small molecules designed to treat neurodegenerative diseases should selectively lower the levels of mHTT but not HTT. However, rapamycin is a classical autophagy inducer, which degrades both HTT and mHTT. However, J3 selectively degrades mHTT, thus a more viable option for promoting mHTT clearance.

**Fig. 7:**
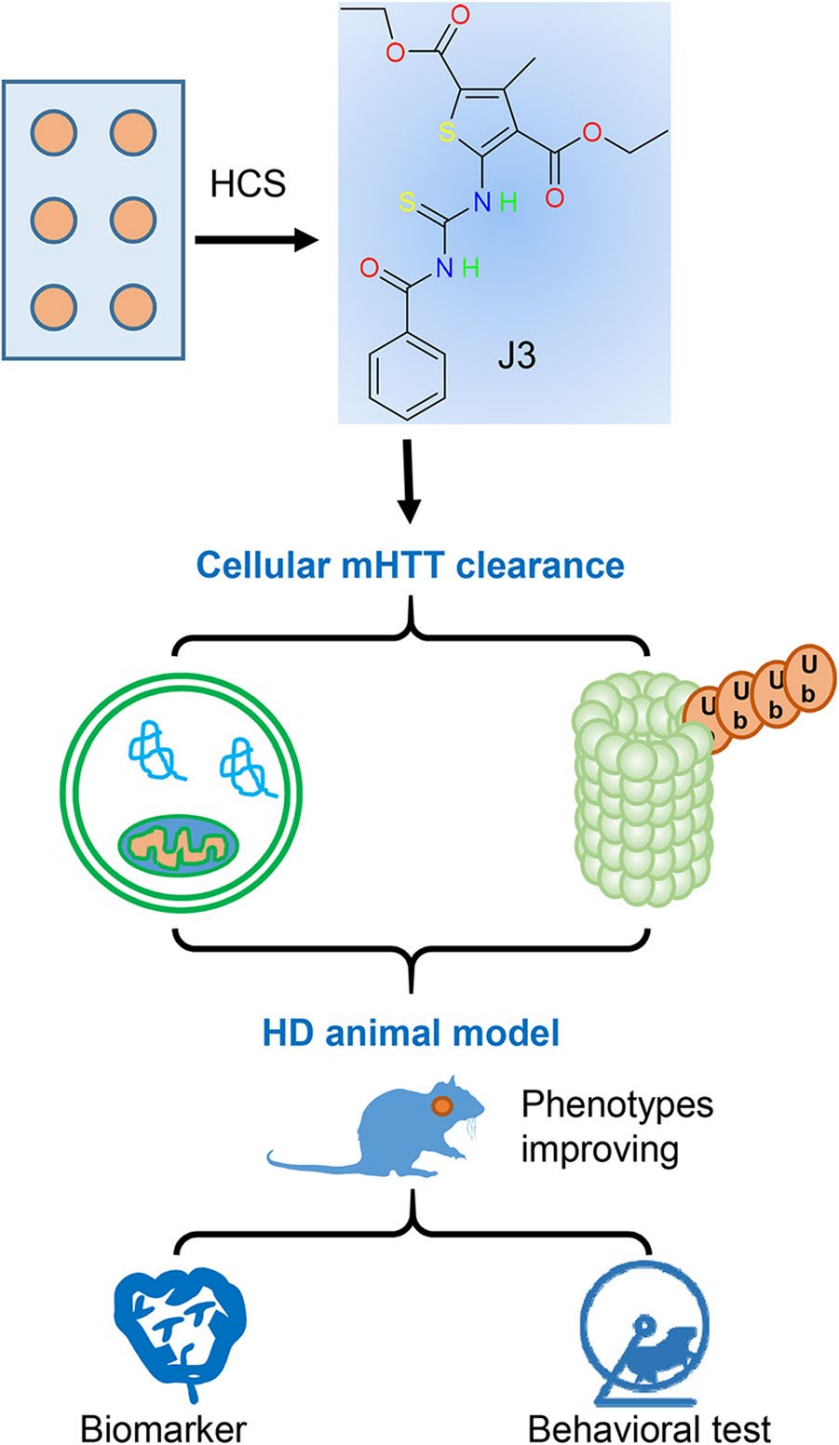
Flow chart of the identification of J3 as a huntingtin cleaner in cellular and in vivo assays

The HdhQ140 knockin mice showed a HD-associated phenotype, characterized by reduced activity at four months, nuclear aggregates in the striatum at six months, and abnormal gait at twelve months [28, 38]. The open-field test, balance beam test, rotarod test, and climbing test showed reduced motor activity in the 12-months-old HdhQ140 mice, consistent with previous studies. However, the HdhQ140 mice showed no cognitive dysfunction. The HdhQ140 mice and WT mice used in this study were not littermates. Therefore, the behavior tests should be interpreted with caution. Behavioral tests for activity and motor functions conducted after administration of J3 revealed that J3 was associated with increased activity and improvement in motor ability. Furthermore, administration of J3 decreased the levels of T-HTT and misfolded proteins in the striatum and increased the levels of the medium spiny neuron marker DARPP-32. The flow chart of this study is illustrated in Fig. 7. Intriguingly, the J3 dose of 60 mg/kg did not show significant differences from the 30 mg/kg dose, suggesting that J3 doses of 30 mg/kg were optimal for treating HD in mice.

J3 possesses good physicochemical properties, such as high lipophilicity and good permeability across the BBB. In addition, J3 could be synthesized via high-yield, high-purity methods. Therefore, J3 offers a promising therapeutic option in neurodegenerative diseases due to its high druggability and enhanced activity.

## Conclusions

This study revealed that J3 induces autophagy through mTOR inhibition. Furthermore, J3 showed a selective ability to clear mHTT through the autophagy and UPS pathways. Moreover, J3 alleviates HD-associated phenotype and increases spontaneous activity and motor function. J3 is a novel promising molecule for the treatment of HD..

## Materials and methods

### Chemicals and antibodies

MG132 (S2619), Chloroquine (CQ, DC661), and Cycloheximide (CHX, S7418) were from Selleckchem (Houston, TX, USA). Rapamycin was from LC Laboratories (Woburn, MA, USA). DMSO (196,055) was from Sigma (St. Louis, MO, USA). Corn oil was from Aladdin (Shanghai, CA). J3 was synthesized as described in Fig. 1P.

Antibodies used in this work were as follows. HTT (MAB2166) and DARPP-32 (AB10518) were from Millipore (Temecula, CA, USA). GFP (sc-9996) and ubiquitin (sc-8017) were from Santa Cruz (Dallas, TX, USA). LC3B (L7543) and β-actin were from Sigma (St. Louis, MO, USA). GAPDH (60,004–1) was from Proteintech (Wuhan, China). ATG4B (M134), ATG5 (M153), ATG16L1 (M150), LC3 (PM036), and p62 (PM045) were from MBL (Woburn, MA, USA). MTOR (2983), p-mTOR (5536), S6K (2708), p-S6K (9208, 9234), ULK1 (4773), and p-ULK1 (5869, 6888) were from Cell Signaling Technology (Danvers, MA, USA). Secondary antibodies conjugated with Alexa Fluor 488 or DyLight 594 secondary antibodies [710, 369, 35, 560] were from ThermoFisher Scientific (Carlsbad, CA, USA).

### Cell culture and plasmid transfection

*ATG5*-deficient MEFs, *ATG4B*-deficient HeLa cells have been described previously [39, 40], ATG16L1-deficient HeLa cells were constructed by Cripsr/Cas9 (sgRNA primers: 5’TCGCGGTGGTTACGCTCGCT3’ #1, 5’ CAGTGTGAGCGGCGCCGGTG 3’ #2). All cells were cultured in DMEM (SH3024301) (Thermo Scientific, Rockford, IL, USA) supplied with 10% (*v*/*v*) fetal bovine serum (FBS, BI, 1752054) and 0.5% Penicillin/Streptomycin (Gibco, Life technologies, 15140163) in 5% (*v*/*v*) CO_2_ incubator at 37℃. HeLa cell stably expressing exon1HTT-103QmCFP (CFP-103Q) was a kind gift from professor Ai Yamamoto (Columbia University) [24]. Cells were cultured in 6-well plates to about 80% density before transfection, then 2 μg of plasmid was transfected to cells by transfection reagent Lipo2000 (Invitrogen, Carlsbad, CA, USA).

### Protein extraction and immunoblot assay

For mouse brain tissue, the mouse striatum was dissected on ice and cut into pieces and sonicated for 20 times at 20% power, and lysed on ice for 1 h in brain lysis buffer (50 mM Tris, 250 mM NaCl, 5 mM EDTA, 1% Triton X-100 pH7.4) plus protease inhibitor cocktail. Then the samples were centrifuged at 16000×*g* at 4 ℃ for 20 min. The supernatant of the striatum sample was collected for further analysis.

The fractionation for resolution of soluble and insoluble Huntingtin in the HD cell model was carried out as previous described [41]. Briefly, cells were lysed on ice and then centrifuged at 15000×*g* for 20 min at 4 °C. The supernatant was collected as the soluble part. The pellet was washed three times by the lysis buffer for 5 min, and the lysis buffer supplemented with 4% SDS was added followed by 30 s sonication which was collected as the insoluble part. The protein concentration of both parts was determined by BCA Protein Quantitation Kit. 30 µg of the soluble part was resolved on 8% SDS-PAGE gels, and 10 µg of the insoluble part was resolved on 3% Tris–acetate polyacrylamide gels for very high molecular mass [42]. Alternatively, another method was applied to simplify the detection of mHTT. In this simplified method, cells were lysed on ice and then centrifuged at 15000×*g* for 20 min at 4 °C. The supernatant was collected as the soluble part. The pellet was washed by buffer containing 2% SDS followed by sonication. After sonication and centrifugation, the supernatant was also transferred to the soluble fraction. The pellet was thought as the insoluble protein, then 5× Loading Buffer was added to both samples and boiled for 5 min. The protein concentration of the soluble part was determined by BCA Protein Quantitation and the insoluble fractions were collected without BCA quantification, but by controlling the number of cells in each group. The soluble and insoluble fractions were loaded separately into 10% SDS-PAGE. These two methods to measure the mHTT were both applied in this study.

For immunoblot, cells were lysed by lysis buffer RIPA (Beyond time, CA) supplied with protease inhibitor cocktail (Bimake, B14012) for 10 min, then the cell lysate was centrifuged for 15 min at 12,000 rpm, 4 ℃, the supernatant was collected and quantified using BCA Protein Quantitation Kit (Thermo, WA319620). Then 30 μg of cell lysate was loaded on 8% or 12% SDS-PAGE gels and transferred to PVDF membranes (Millipore, ISEQ00010). After blocking by 5% nonfat milk and incubation with primary and secondary antibodies, specific proteins were detected using High-sig ECL Western Blotting Substrate (Tanon, 180–5001). Images were captured by Tannon Image Station 4000.

### Immunostaining analysis

For cell samples, cells were cultured at 24-well confocal plates, after washing by PBS for two times, and fixed by 4% paraformaldehyde (PFA, pH = 7.4) for 15 min at room temperature, then permeabilized by 0.1% Triton X-100 for 15 min. The samples were blocked with goat serum for 30 min. Primary antibodies were added and incubated at 4 °C overnight, then followed by secondary antibodies for another 1 h at room temperature.

For mouse brain slices, mice were anesthetized and sacrificed, then perfused with 4% PFA for 48 h at 4 °C. Then the tissues were incubated in 15% sucrose for 24 h and then in 30% sucrose for 48 h at 4 °C and sectioned into 30 μm thick slices. The subsequent experiments were performed similarly to the cell experiment mentioned above except using 5% BSA for blocking. Primary antibodies HTT (1:200), DARPP-32 (1:5000), Ub (1:200) were applied followed by incubation with secondary antibodies. Images of above samples were captured by EVOS FL Auto (Life Technologies, Bothell, WA, USA) and analyzed blindly by Image J for aggregates quantifications. Fluorescence images were also taken by EVOS FL Auto or Olympus confocal microscopes (FV3000). For manual quantification of the puncta formation, at least 3 optical fields with more than 50 cells per experimental condition were analyzed. GFP-LC3 dots of cells were counted from different groups. Data from repeated experiments were subjected to statistical analysis.

### Long-lived protein degradation

Long-lived protein degradation assay was carried out as described previously [43], Briefly, MEFs were cultured in 24-well plates overnight, L-[^14^C]-valine (PerkinElmer, NEC291EU050UC) was added to a final concentration of 0.2 µCi/ml to label intracellular proteins. Cells were incubated for 18–24 h before changing to fresh medium for another hour with 10% cold l-valine to deplete labeled short-lived proteins. The cells were then incubated in EBSS or DMEM (plus 0.1% of BSA and 10 mM of valine) with or without J3 for an additional 6 or 16 h. The culture medium was recovered, from which the degraded long-lived proteins were measured via liquid scintillation.

### J3 synthesis

Benzoyl isothiocyanate (0.82 g, 5.0 mmol) and diethyl 5-amino-3-methylthiophene-2,4-dicarboxylate (1.54 g, 6.0 mmol) were dissolved in anhydrous MeCN (30 mL) and stirred at reflux. After completion of the reaction, monitored by thin layer chromatography (TLC), it was then cooled to room temperature and evaporated under vacuum. The residue was purified by column chromatography to afford diethyl 5-(3-benzoylthioureido)-3-methy-lthiophene-2,4-dicarboxylate (1.47 g, mmol, 70.0% yield) as a yellow powder. ^1^H NMR (400 MHz, CDCl3) δ 14.93 (s, 1H), 9.23 (s, 1H), 8.07 – 7.89 (m, 2H), 7.67 (t, J = 7.4 Hz, 1H), 7.56 (t, J = 7.7 Hz, 2H), 4.55 (q, J = 7.1 Hz, 2H), 4.37 (q, J = 7.1 Hz, 2H), 2.83 (s, 3H), 1.44 (dt, J = 19.4, 7.1 Hz, 6H). **ESI–MS** *m/z*: 421.2 [M + H]^+^_._

### Mouse model and genotyping

Huntington’s disease knock-in mouse model expression 140Q (Hdh^Q140^) knock-in mice were kindly gifted from Professor Marian Difiglia (MassGeneral Hospital). The generation and characterization of Hdh140Q knock-in mice have been described previously [38]. Specifically, the mice’s genetic background was derived from C57BL/6, and exon1 of mice HTT was replaced by human HTT that containing 140 CAG repeats. The homozygous of Hdh^Q140^ knock-in mice were mating with wild-type mice in male: female ration of 1:2. The first generation was heterozygotes and mating between littermates male and female mice to generate homozygous and wild-type mice. After genotyping, mice were kept for corresponding months. Homozygous mice were selected after 4 generations of Huntington’s disease mice cross the same genetic background. Mice were group-housed by 5 adults per cage with 12 h light/dark cycle. The mouse experiments were carried out following the general guidelines published by the Association for Assessment and Accreditation of Laboratory Animal Care. The Animal Care and Use Committee of the School of Medicine at Guangzhou University of Chinese medicine approved the protocol used in animal experiments (No.20190704006). Mouse brain tissue was acutely dissected for protein extraction and immunohistology analysis.

When the mice grew to 6 weeks, 2–3 mm of mouse tail was cropped and collected in tubes. After extraction of DNA, PCR reaction buffer was prepared with primers (F: 5′ CTGCACCGACCGTGAGTCC 3′, R: 5′ GAAGGCACTGGAGTCGTGAC 3′), then the amplification system was operated and separated by 2% agarose. Under gel imaging system, two bands appeared at about 250 bp and 170 bp were thought as wild-type and homozygous mouse, respectively. While heterozygotes had both two bands.

### Mouse grouping and administration

After genotyping, mice were kept to 12 months. Hdh^Q140^ mouse appeared less activity, locomotor deficient, and gait abnormal at 12 months. All mice were operated behavioral experiments grouped by gene type and the abnormal mice were excluded at 12 months. Having finished the pilot test, the formal protocol was conducted. J3 was dissolved and distributed in corn oil to 12 mg or 24 mg/mL, mice were intraperitoneal injection of two day’s dosage of J3 suspension 0.1 mL per 20 g and 3 times per week.

### Mouse behavioral experiments

In the behavioral experiment, the gene types and grouping message of all mice were blind and all experiments were conducted in the light phase. Both male and female mice had been used, between two mice to be tested, 75% alcohol was prepared to eliminate substances and smell that left in machine or chamber.

For the activity test, mice were placed in the dark chamber (110 mm × 100 mm × 110 mm) for 5 min, the rearing number indicated the total number of events that the mice lifted up at least two limbs. The counts of rearing numbers were calculated by machine automatically and showed on the screen of the machine.

For open-field tests, mice were placed in a blue chamber (110 mm × 100 mm × 110) mm in the behavioral test room, and locomotion was captured by a camera on top of the chamber and recorded for 15 min. The times of event that the mouse lifting its forelimbs, traveling tract and distance were then analyzed by the behavioral analysis system.

Based on the animal’s nature to explore a novel object more than a familiar object, novel object recognition (NOR) was designed to test the ability of distinguishing objects. Mice were placed in the testing room and stayed for 1 h to reduce stress, experimental animals were briefly handled for 3 days. On day 1, animals were habituated in the empty open field for 30 min to lower basal stress levels. On day 2, animals were presented with two identical cylinders (A1 and A2) in the chamber for 5 min to finish the training session. The cylinders were placed opposite each other 10 cm from each wall. After 24 h, in the NOR test, animals were exposed to a familiar object (A2) and a novel object (B). The novel object was a cube that had a different size to the familiar object. A2 and B were placed in the same locations as A1 and A2 were placed in the training sessions. Object exploration was measured by the stopwatch, and ‘exploration’ was defined as sniffing or touching the object with the nose. Behavior was not scored as ‘exploration’ when the animal was using the object to rear up or when the animal was sitting on the object. A percent of time spent exploring the novel object (TN) relative to the total time spent exploring both objects (TN + TF, TF refers to the time devoted to the familiar object) can be a measure of novel object recognition. Data are expressed as a recognition index (RI): RI = TN/ (TN + TF).

For the balance beam test, mice were pre-trained to across the balance beam that settled at 30-degree incline cradle in one-way. According to the reference, beams could be 80 cm long, 15 mm or 10 mm diameter smooth round one or 15 mm or 10 mm side cube beams. In the training and experimental session, the mice were started at the lower end to climb. At the end of the inclined beam, there is a 20 × 20 cm wooden platform. A 20 × 20x10 cm cage with a 5 × 5 cm door towards the higher end of beams was set to attract mouse walk into the little house. Having trained the mouse to climb the balance beam in single way, the number of slip faults was counted to evaluate the balance and coordination skills.

For the rotarod test, mice were pre-trained on 3 consecutive days on the rotarod rotating at 4 rpm for 2 min. Mice were then tested for 3 days at 4 rpm, 8 rpm or accelerating speed ranging from 4 to 8 rpm within 2 min. Each performance was recorded as the time in seconds spent on the rotating rod until falling off or until the end of the task. Each test including three repetitions with an inter-trial interval of 1 h to reduce stress and fatigue, and the means from these three runs were analyzed for each mouse.

For the climbing test, a vertical 1 cm diameter smooth metal rod was fixed in the platform. In the training session, mice were placed at the top of the rod, once the mice scratched the rod steadily, the mice climb down along the rod autonomously. The time spent on the climbing that mice across between the top and the platform. The scores of motor evaluation of mice were noted. If the mice slide along the rods step by step, count 0 point; if mice slip length less than 20 cm, count 0.5 points; if mice could not scratch firmly to slip along the rod, count 1 point; if mice could not scratch the rod and drop, count 1.5 points. Each test included three repetitions with an inter-trial interval of 1 h to reduce stress and fatigue, and the means from these three runs were analyzed for each mouse.

### Immunohistochemistry

The pretreatment of immunohistochemistry is similar to the immunostaining process. The brain tissue was fixed and cut into thick slices followed by blocking by 5% BSA and incubation with primary antibodies HTT (1:100) and DARPP-32 (1:3000) at 4 ℃ overnight. Then the samples were incubated with secondary antibodies for 30 min at room temperature and developed by DAB agent. Images were captured by EVOS microscopes and analyzed blindly by Image J for quantifications.

### Blood–brain barrier measurement

The blood–brain barrier of J3 was investigated in 10-week-old mice following a single intravenous injection (IV) at 6 mg/kg. J3 was prepared in 10% DMA + 20% Solutol + 70% (20%SBE-β-CD in Saline) to get the required solution for IV. Plasma and brain collection intervals of post-dose for IV is at 0.5 h. 3 male ICR mice of SPF were used in each group. In brief, blood samples taken via submandibular vein were placed in tubes containing K2-EDTA and centrifuged at 6800 g for 6 min at 2–8 °C within 1 h. Samples were stored frozen at approximately −80 °C. The brain of mice was collected after the animals were euthanized by CO_2_ inhalation. The ratio of brain-plasma concentration was calculated by Conc. in brain/ Conc. in plasma.

### Statistic assay

All data were derived from three independent experiments. Statistical comparisons between two groups were conducted by the unpaired two-tailed student tests. Statistical comparisons among multiple groups were conducted by one-way ANOVA tests, and Kruskal–Wallis test was applied between groups to be compared when performing statistics in GraphPad Prism 8.0. When the p value was less than 0.05, there was a significant difference between groups.

## Supplementary Information

**Additional file 1: Figure S1.** (A) WT-HeLa cells were treated with 20 μM of J3 for 3, 6, and 12 h for western blot, quantification of the protein expression of p62 (B) and LC3 (C) was analyzed. (D) GFP-LC3-HeLa were treated as (A), GFP-LC3 dots were analyzed. Scale bar = 20 μm. (E) GFP-LC3 dots of (D) were quantified. (F) GFP-LC3-MEF and GFP-LC3-A549 cells were incubated with 20 μM of J3 for 6 h. GFP-LC3 dots were analyzed. Scale bar = 20 μm. (G) Quantification of GFP-LC3 dots in (F) was performed. (H) U251 cells were treated with 20 μM of J3 with or without 40 μM of CQ for 6 h for western blot, quantification of the protein expression of p62 (I) and LC3 (J) was analyzed. (K) HeLa cells were treated with CM and starvation (EBSS) with or without 5 mM of 3-MA, 1 μM of Rap, and 10 μM of J3 for 6 h. Percentages of long-lived protein degradation were analyzed. Data are presented as mean ± sem from three individual experiments or three different fields. *p < 0.05, **p < 0.01, ***p < 0.001.

**Additional file 2: Figure S2.** WT-HeLa cells were incubated with 20 μM of J3 with or without 2 mM of NAC, 1 μM of compound C, 10 μM of U0126, 10 μM of BAPTA for 6 h, respectively. The protein level of LC3-II was detected by western blot and quantified.

**Additional file 3: Figure S3.** (A) CFP-103Q-HeLa cells were treated with 20 μM of J3 for 12, 24, 48 h, respectively. The insoluble (I-mHTT), soluble mHTT (S-mHTT) and LC3 were detected for western blot. (B-C) The quantification of insoluble (B) and soluble mHTT (C) at different times was analyzed. (D) CFP-103Q-HeLa were treated with 5, 10, and 20 μM of J3 for 48 h, the protein level of mHTT and LC3 were measured by western blot. (E–F) The quantification of insoluble (E) and soluble mHTT (F) treated by different doses of J3 was analyzed. Data are presented as mean ± sem from three individual experiments. *p < 0.05, **p < 0.01.

**Additional file 4: Figure S4.** (A-C) CFP-103Q-HeLa cells were treated with 20 μM of J3 for 48 h. 10 μM of CHX was added at the last 6 h. The protein expression of mHTT (anti-GFP, sc-9996) was detected by western blot (A), and the quantification of insoluble mHTT (B) and soluble mHTT (C) was analyzed. (D-F) CFP-103Q-HeLa cells were treated with 20 μM of J3 for 48 h. 40 μM of CQ was added at the last 6 h. The protein expression of mHTT (anti-GFP, sc-9996) was detected by western blot (D), and the quantification of insoluble mHTT (E) and soluble mHTT (F) was analyzed. (G-I) CFP-103Q-HeLa cells were treated with 20 μM of J3 for 48 h. 10 μM of MG132 was added at the last 6 h. The protein expression of mHTT (anti-GFP, sc-9996) was detected by western blot (G), and the quantification of insoluble mHTT (H) and soluble mHTT (I) was analyzed. Data are presented as mean ± sem from three individual experiments. *p < 0.05, **p < 0.01, ***p < 0.001, ns means not significant.

**Additional file 5: Figure S5.** (A) The flow diagram of the mice generation, genotype identification, and the administration methods (Hdh^Q140/Q140^ and WT mice are not littermates in our study). (B) Dissection of the mice striatum were shown in the picture, the red line indicates the outline for a piece of striatum. (C) The immunostaining of T-HTT (anti-HTT, MAB2166) in the striatum of WT and homozygous mice was analyzed at 12-month-old. (D) The expression of total mHTT (anti-HTT, MAB2166) and DARPP-32 in the striatum of WT and heterozygote Hdh.^Q140/−^ mice and the relative protein level was analyzed at 12-month-old. Data are presented as mean ± SD from three individual samples. *p < 0.05, **p < 0.01.

**Additional file 6:** **Figure S6.** (A-B) Analysis of the expression level of T-HTT (anti-HTT, MAB2166) and DARPP32 in the striatum of 15-month-old homozygous Hdh^Q140/Q140^ mice with or without 60 mg/kg of J3 administration for 3 months. Data are presented as mean ± sem from three individual samples. *p < 0.05, **p < 0.01, ***p < 0.001. (C) Values of parameters characterizing physicochemical and basic ADME properties calculated using ACD/Percepta.

## Abbreviations

AMPK: Adenosine 5′monophosphate activated protein kinase
ATG: Autophagy-related gene
BBB: Blood–brain barrier
CNS: Central nervous system
CFP: Cyan fluorescent protein
CHX: Cycloheximide
CQ: Chloroquine
DARPP-32: Dopamine- and cAMP-regulated neuronal phosphoprotein 32 kDa
DMSO: Dimethyl sulfoxide
FBS: Fetal bovine serum
GFP: Green fluorescent protein
HD: Huntington’s disease
HTT: Huntingtin
KO: Knock-out
K: Lys
LC3: Microtubule-associated protein 1 light chain 3
mHTT: Mutant Huntingtin
mTOR: Mammalian target of rapamycin
MAPK: Mitogen-activated protein kinase
NAC: N-acetyl-L-cysteine
PBS: Phosphate buffer saline
PI3K: Phosphatidylinositol 3-kinase
PROTAC: Proteolysis targeting chimera
PVDF: Polyvinylidene difluoride
ROS: Reactive oxygen species
SDS: Sodium dodecyl sulfate
Ub: Ubiquitin
ULK1: Unc-51-like kinase 1
UPS: Ubiquitin–proteasome system
