# Supplementary figures and images for: Discovery of an autophagy inducer J3 to lower mutant huntingtin and alleviate Huntington’s disease-related phenotype
Source: Cell Biosci. 2022 Oct 8;12:167. doi: 10.1186/s13578-022-00906-3 (PMC9548129; doi:10.1186/s13578-022-00906-3)

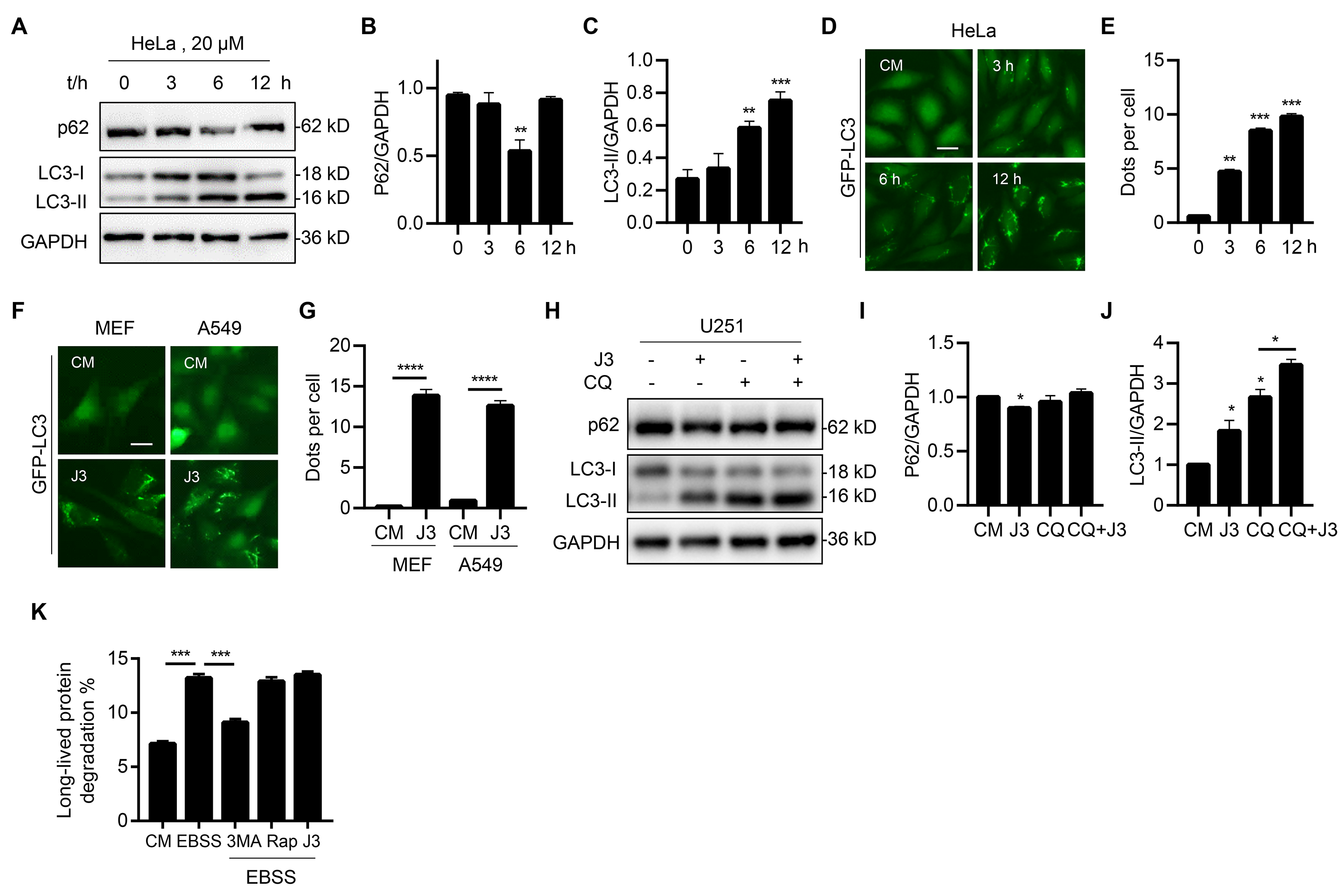

Supplement: Supplementary file 1 — Additional file 1: Figure S1. (A) WT-HeLa cells were treated with 20 μM of J3 for 3, 6, and 12 h for western blot, quantification of the protein expression of p62 (B) and LC3 (C) was analyzed. (D) GFP-LC3-HeLa were treated as (A), GFP-LC3 dots were analyzed. Scale bar = 20 μm. (E) GFP-LC3 dots of (D) were quantified. (F) GFP-LC3-MEF and GFP-LC3-A549 cells were incubated with 20 μM of J3 for 6 h. GFP-LC3 dots were analyzed. Scale bar = 20 μm. (G) Quantification of GFP-LC3 dots in (F) was performed. (H) U251 cells were treated with 20 μM of J3 with or without 40 μM of CQ for 6 h for western blot, quantification of the protein expression of p62 (I) and LC3 (J) was analyzed. (K) HeLa cells were treated with CM and starvation (EBSS) with or without 5 mM of 3-MA, 1 μM of Rap, and 10 μM of J3 for 6 h. Percentages of long-lived protein degradation were analyzed. Data are presented as mean ± sem from three individual experiments or three different fields. *p < 0.05, **p < 0.01, ***p < 0.001. [file 13578_2022_906_MOESM1_ESM.tif]

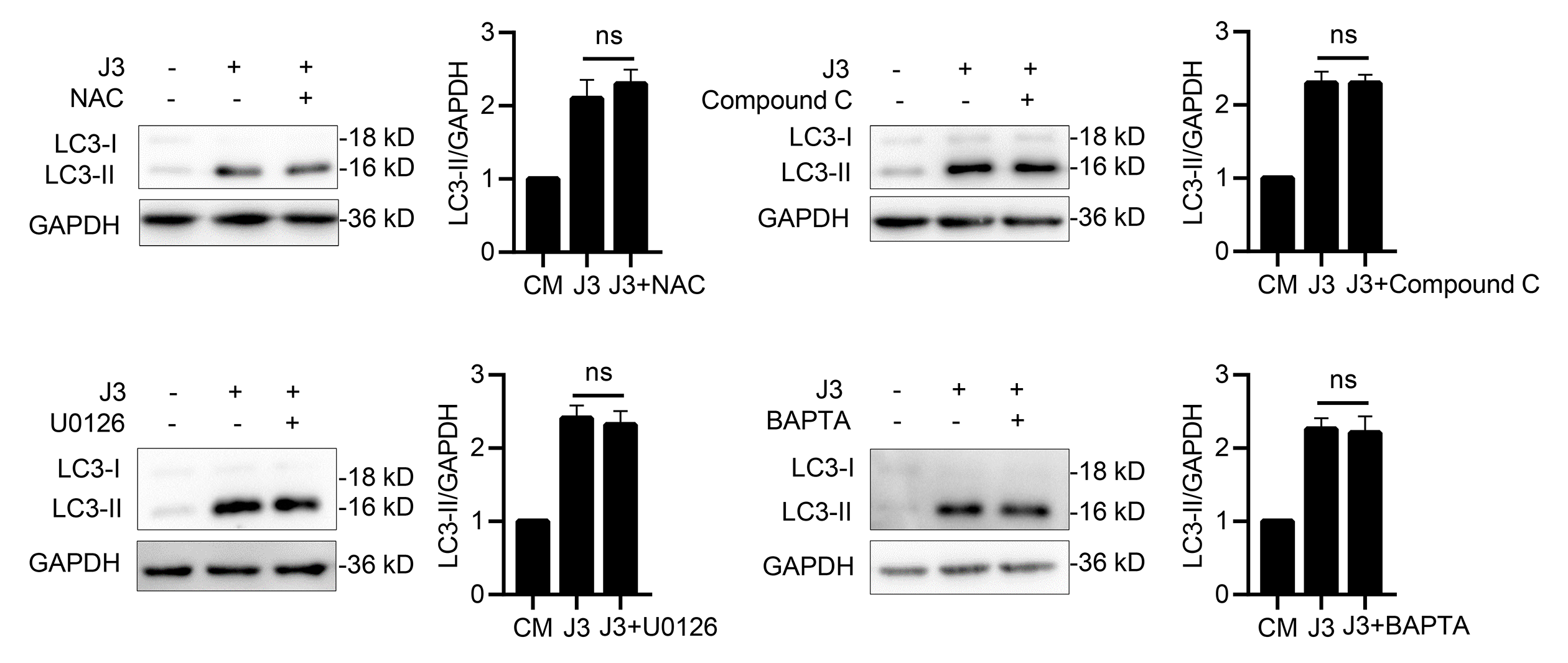

Supplement: Supplementary file 2 — Additional file 2: Figure S2. WT-HeLa cells were incubated with 20 μM of J3 with or without 2 mM of NAC, 1 μM of compound C, 10 μM of U0126, 10 μM of BAPTA for 6 h, respectively. The protein level of LC3-II was detected by western blot and quantified. [file 13578_2022_906_MOESM2_ESM.tif]

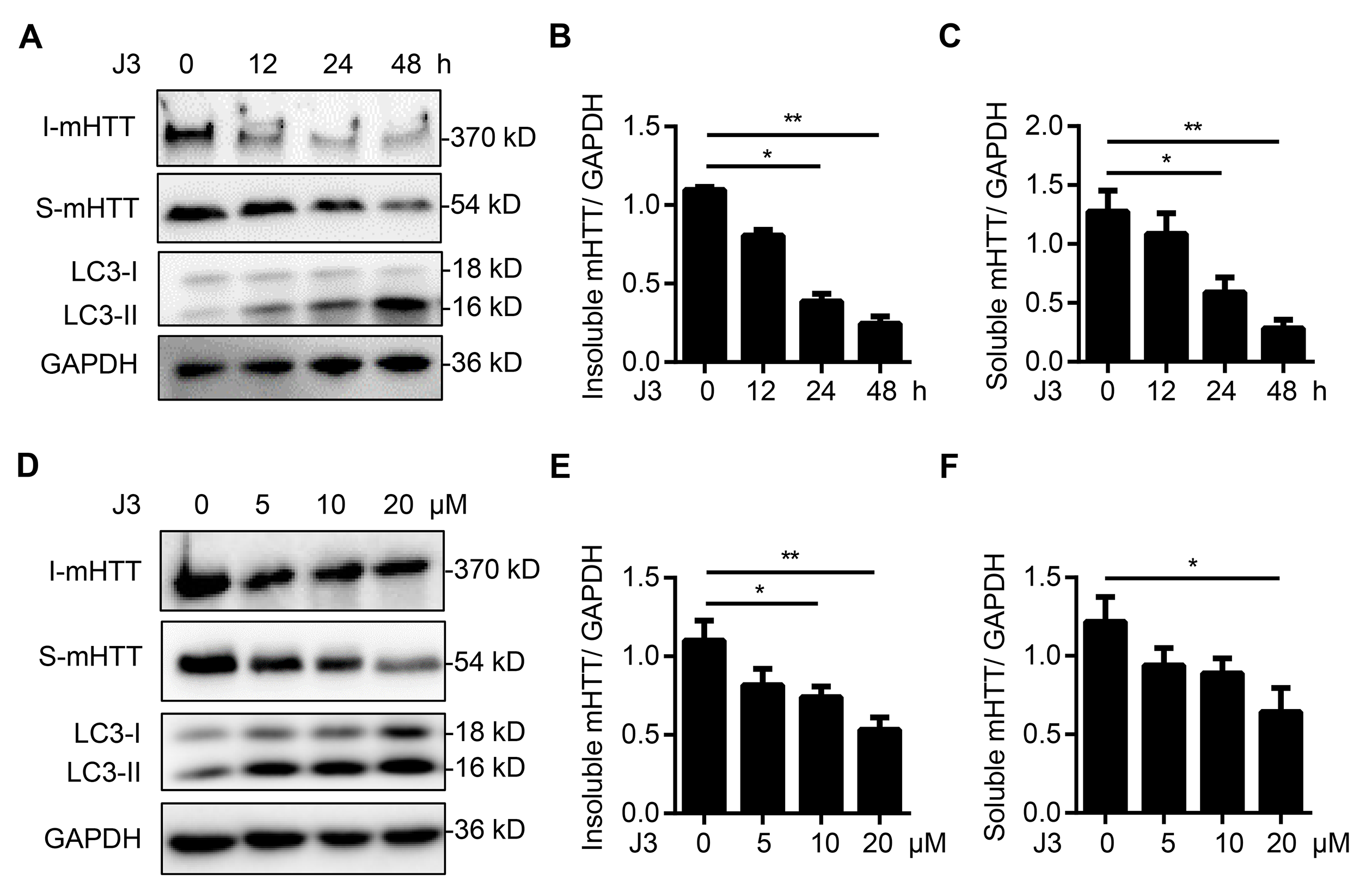

Supplement: Supplementary file 3 — Additional file 3: Figure S3. (A) CFP-103Q-HeLa cells were treated with 20 μM of J3 for 12, 24, 48 h, respectively. The insoluble (I-mHTT), soluble mHTT (S-mHTT) and LC3 were detected for western blot. (B-C) The quantification of insoluble (B) and soluble mHTT (C) at different times was analyzed. (D) CFP-103Q-HeLa were treated with 5, 10, and 20 μM of J3 for 48 h, the protein level of mHTT and LC3 were measured by western blot. (E–F) The quantification of insoluble (E) and soluble mHTT (F) treated by different doses of J3 was analyzed. Data are presented as mean ± sem from three individual experiments. *p < 0.05, **p < 0.01. [file 13578_2022_906_MOESM3_ESM.tif]

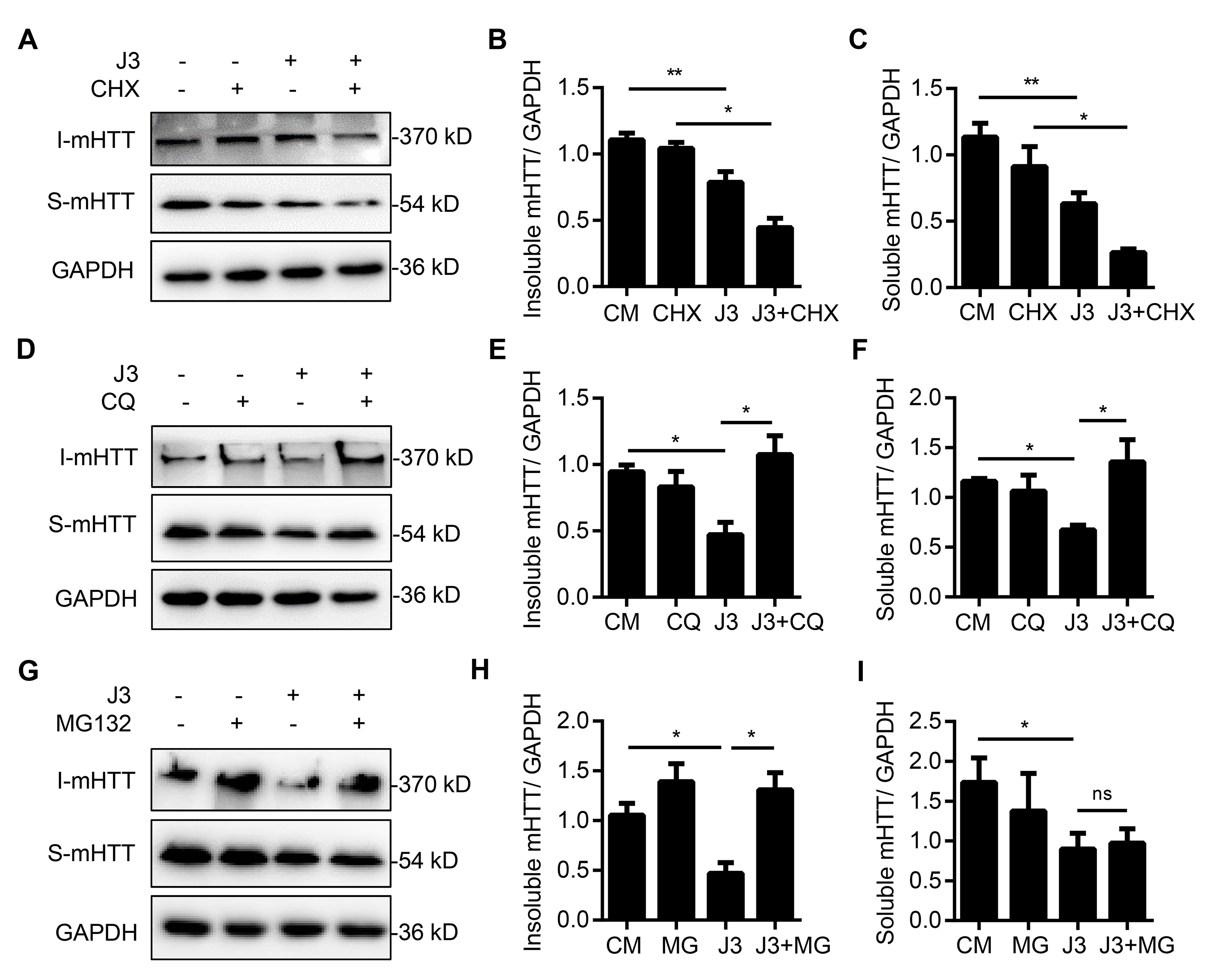

Supplement: Supplementary file 4 — Additional file 4: Figure S4. (A-C) CFP-103Q-HeLa cells were treated with 20 μM of J3 for 48 h. 10 μM of CHX was added at the last 6 h. The protein expression of mHTT (anti-GFP, sc-9996) was detected by western blot (A), and the quantification of insoluble mHTT (B) and soluble mHTT (C) was analyzed. (D-F) CFP-103Q-HeLa cells were treated with 20 μM of J3 for 48 h. 40 μM of CQ was added at the last 6 h. The protein expression of mHTT (anti-GFP, sc-9996) was detected by western blot (D), and the quantification of insoluble mHTT (E) and soluble mHTT (F) was analyzed. (G-I) CFP-103Q-HeLa cells were treated with 20 μM of J3 for 48 h. 10 μM of MG132 was added at the last 6 h. The protein expression of mHTT (anti-GFP, sc-9996) was detected by western blot (G), and the quantification of insoluble mHTT (H) and soluble mHTT (I) was analyzed. Data are presented as mean ± sem from three individual experiments. *p < 0.05, **p < 0.01, ***p < 0.001, ns means not significant. [file 13578_2022_906_MOESM4_ESM.tif]

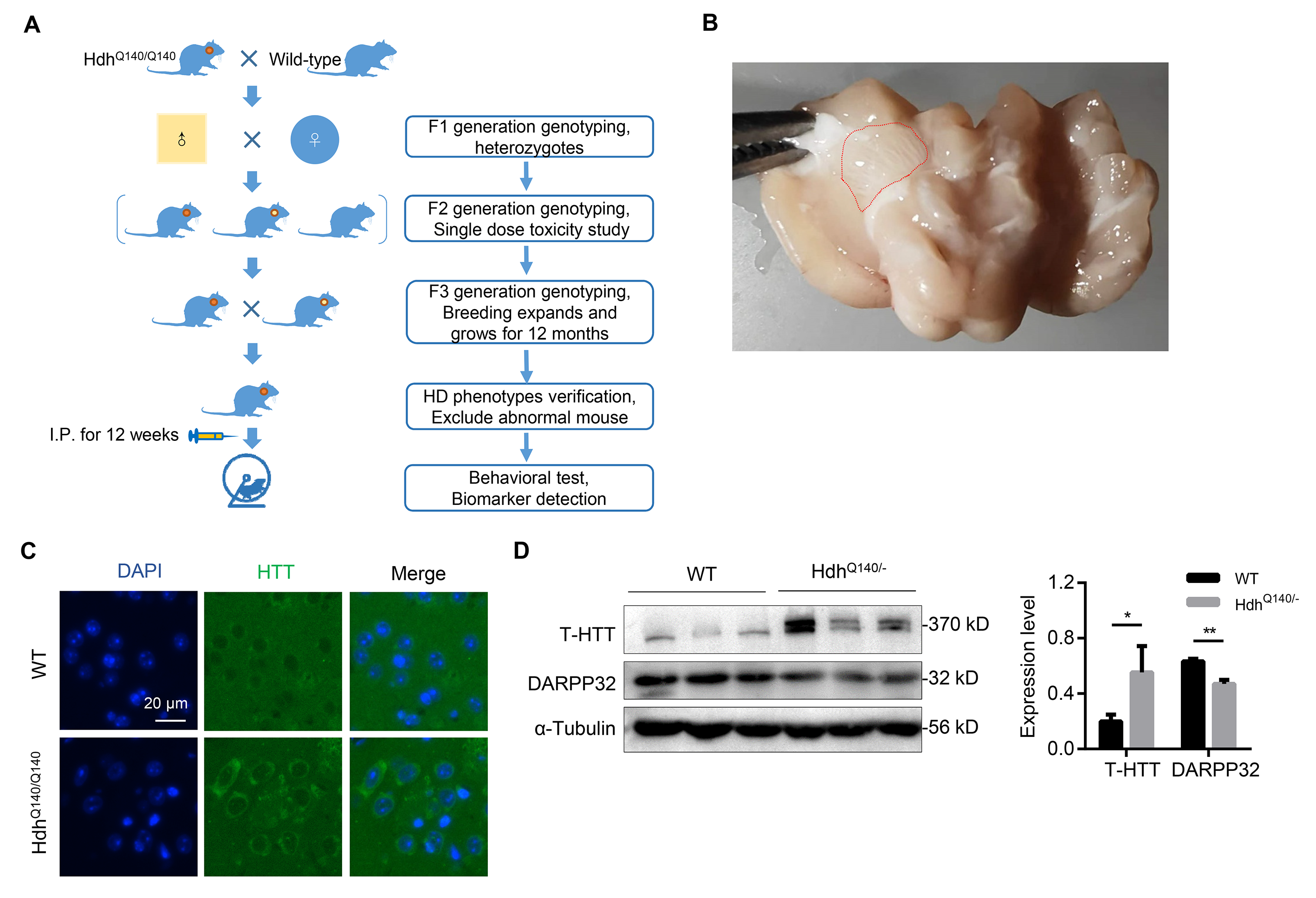

Supplement: Supplementary file 5 — Additional file 5: Figure S5. (A) The flow diagram of the mice generation, genotype identification, and the administration methods (HdhQ140/Q140 and WT mice are not littermates in our study). (B) Dissection of the mice striatum were shown in the picture, the red line indicates the outline for a piece of striatum. (C) The immunostaining of T-HTT (anti-HTT, MAB2166) in the striatum of WT and homozygous mice was analyzed at 12-month-old. (D) The expression of total mHTT (anti-HTT, MAB2166) and DARPP-32 in the striatum of WT and heterozygote Hdh.Q140/− mice and the relative protein level was analyzed at 12-month-old. Data are presented as mean ± SD from three individual samples. *p < 0.05, **p < 0.01. [file 13578_2022_906_MOESM5_ESM.tif]

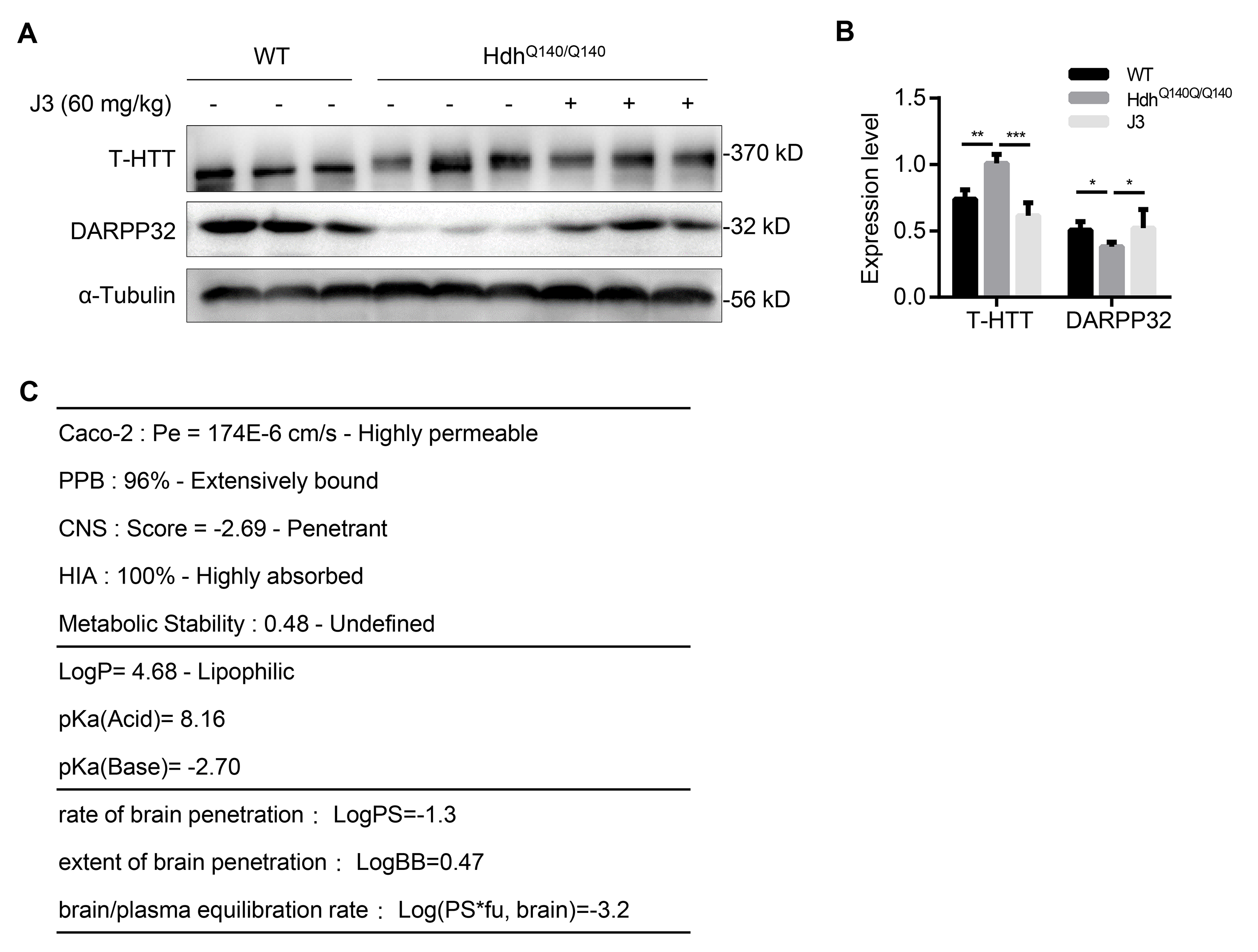

Supplement: Supplementary file 6 — Additional file 6: Figure S6. (A-B) Analysis of the expression level of T-HTT (anti-HTT, MAB2166) and DARPP32 in the striatum of 15-month-old homozygous HdhQ140/Q140 mice with or without 60 mg/kg of J3 administration for 3 months. Data are presented as mean ± sem from three individual samples. *p < 0.05, **p < 0.01, ***p < 0.001. (C) Values of parameters characterizing physicochemical and basic ADME properties calculated using ACD/Percepta. [file 13578_2022_906_MOESM6_ESM.tif]
